# Genome-wide screening and characterization of long noncoding RNAs involved in flowering/bolting of *Lactuca sativa*

**DOI:** 10.1186/s12870-022-04031-8

**Published:** 2023-01-02

**Authors:** Aboozar Soorni, Marzieh Karimi, Batoul Al Sharif, Khashayar Habibi

**Affiliations:** 1 Zarinshahr, Isfahan, Iran; 2 Department of Biotechnology, College of Agriculture, Isfahan University of Technology, Isfahan, Iran

**Keywords:** Bolting, Long non-coding RNA, Lettuce, Regulation

## Abstract

**Background:**

Lettuce (*Lactuca sativa* L.) is considered the most important vegetable in the leafy vegetable group. However, bolting affects quality, gives it a bitter taste, and as a result makes it inedible. Bolting is an event induced by the coordinated effects of various environmental factors and endogenous genetic components. Although bolting/flowering responsive genes have been identified in most sensitive and non-sensitive species, non-coding RNA molecules like long non-coding RNAs (lncRNAs) have not been investigated in lettuce. Hence, in this study, potential long non-coding RNAs that regulate flowering /bolting were investigated in two lettuce strains S24 (resistant strain) and S39 (susceptible strain) in different flowering times to better understand the regulation of lettuce bolting mechanism. For this purpose, we used two RNA-seq datasets to discover the lncRNA transcriptome profile during the transition from vegetative to reproductive phase.

**Results:**

For identifying unannotated transcripts in these datasets, a 7-step pipeline was employed to filter out these transcripts and terminate with 293 novel lncRNAs predicted by PLncPRO and CREMA. These transcripts were then utilized to predict cis and trans flowering-associated targets and Gene Ontology (GO) and Kyoto Encyclopedia of Genes and Genomes (KEGG) pathway enrichment analysis. Computational predictions of target gene function showed the involvement of putative flowering-related genes and enrichment of the floral regulators *FLC*, *CO*, *FT*, and *SOC1* in both datasets. Finally, 17 and 18 lncRNAs were proposed as competing endogenous target mimics (eTMs) for novel and known lncRNA miRNAs, respectively.

**Conclusion:**

Overall, this study provides new insights into lncRNAs that control the flowering time of plants known for bolting, such as lettuce, and opens new windows for further study.

**Supplementary Information:**

The online version contains supplementary material available at 10.1186/s12870-022-04031-8.

## Background

Lettuce *(Lactuca sativa* L.) is an annual and leafy vegetable belonging to the Asteraceae family [1, 2]. It is considered of great importance as it contributes to human nutrition. Lettuce is rich in fiber, minerals, flavonoids, chlorophyll, and carotenoids and is widely used as a raw vegetable in salads, sandwiches, wraps, or cooked [3, 4]. For these reasons, it is cultivated in many countries in addition to being widely grown in some home gardens. China, U.S., Spain, Italy, India, and Japan are the leading producers; however, the cultivation of *Lactuca* is accompanied by several problems that could decrease its productivity and quality [3]. One of these undesirable traits in leafy lettuce is bolting.

Bolting is the fast transition from the vegetative to the reproductive period which characterizes a fundamental step in the development of flowering plants [5]. During this, the vegetative phase ends so quickly to be followed by rapid floral stem elongation and bud differentiation, thus initiating the reproductive stage [6]. This phenomenon is controlled by environmental as well as genetic factors that determine the flowering time. External conditions mainly involve temperature, light intensity, and light exposure duration [5]. In fact, more light is shed on investigating the genetic regulatory mechanisms that control bolting and other agronomic traits, mainly those affecting the quality of crops [7]. Genetically speaking, genes, transcriptomes, and proteins are all players that are molecularly involved in intercalated regulation pathways. According to findings, five major flowering pathways including vernalization, photoperiod, autonomous, aging, and phytohormone especially gibberellin (GA) pathways along with circadian clock, ambient temperature, development, and FRIGIDA (FRI)-dependent pathways have been determined to control bolting and flowering time [8]. Around 300 genes found in *Arabidopsis thaliana* are involved in controlling flowering, for example, FLOWERING LOCUS T (*FT*), SUPPRESSOR OF OVEREXPRESSION OF CO 1 (*SOC1*), and LEAFY (*LFY*) serve as main contributors to determine the inflorescence time [9]. Furthermore, two genes, *LsFT* and *LsSOC1*, are known to intervene in the heat-induced bolting process. Expression of *LsFT* can be promoted by heat treatment, while its knockdown delays bolting, and in consequence plants fail to respond to high temperatures [3]. *LsSOC1* also acts by activating bolting upon high temperatures. In addition, MADS-box genes and GAs can as well regulate bolting in lettuce [10].

What is still slightly ambiguous, is the putative roles of non-coding genes. Transcripts of non-coding genes have revealed regulatory functions in different pathways affecting flowering and the reproductive switch [11]. One of these types of transcripts is the long non-coding RNA known as lncRNA. LncRNAs are non-coding RNAs that are largely heterogeneous accounting for a length equal or greater than 200 nucleotides. They are similar to coding mRNA in being transcribed by RNA Pol II and acquiring a poly-adenylated tail [12]; however, they are not translated into proteins. LncRNAs originate from intergenic regions or the intronic and exonic regions of the coding genes [11], and they usually regulate the expression of other genes and act as miRNA sequesters to allow the expression of mRNAs targeted by miRNA.

Various biological processes; development (from pollen to roots), reproduction (flowering/bolting), and stress responses are under the control of lncRNAs which indicate their fundamental regulatory roles [13]. Functional analysis of lncRNAs has also discovered the potential roles of lncRNAs in regulating the flowering/bolting time. For example, the lncRNAs COLDAIR [14], COLDWRAP [15], and COOLAIR [16] are transcribed from *FLC* and function in *FLC* epigenetic silencing. COOLAIR functions in the regulation of flowering in *Arabidopsis* in both vernalization and autonomous pathways by modulating *FLC* expression through multiple mechanisms [16]. COLDAIR and COLDWRAP also function in the regulation of flowering through the vernalization pathway [14, 15]. COLDAIR acts by associating with Polycomb group proteins to mediate silencing of *FLC* and by affecting chromatin looping at *FLC* in response to vernalization, while COLDWRAP coordinates vernalization-mediated Polycomb silencing of the *FLC* in addition to forming intragenic chromatin loop that represses *FLC* [14]. Research has also identified the Antisense Long (ASL) transcript being involved in early-flowering *Arabidopsis*, specifically those that do not need vernalization to flower [17]. Although ASL is alternatively spliced, it is not polyadenylated, contrary to other lncRNAs transcribed from *FLC*. Its length is more than 2000 nucleotides, starting at the same promoter as COOLAIR but transcribed from the antisense strand. It is true that both COOLAIR and ASL 5′ regions overlap; however, ASL spans intron 1, which is prominent for keeping *FLC* silenced, and includes the COLDAIR region which is transcribed in the sense direction. By physical contact, the ASL transcript collaborates with the FLC locus and H3K27me3, suggesting that ASL and COOLAIR perform distinctly in *FLC* silencing and expectedly in the maintenance of H3K27me3 [17]. ASL functions in the regulation of flowering in the autonomous pathway in Arabidopsis. AtRRP6L adjusts ASL to balance H3K27me3 levels [17, 18]. Other findings have discovered a flowering associated intergenic lncRNA (*FLAIL*) that suppresses flowering in *Arabidopsis.* Knock-down of FLAIL RNA levels has led to early flowering along with genomic mutations, revealing the trans-acting role of FLAIL RNA in restraining the flowering of *Arabidopsis* by interfering in transcriptionally regulating genes directly bound by FLAIL [19].

An overall review of the RNA section reveals that the lncRNAs are detected and assessed through high throughput sequencing, and bioinformatics approaches [20]. Similarly, several publications have identified lncRNAs implicated in bolting. Ghorbani et al. [11] performed transcriptome and qPCR analyses to reveal lncRNAs and hub genes associated with spinach bolting in two accessions. Correspondingly, Tu et al. [21] identified the miRNA-lncRNA-TF regulatory networks attributed to leaf and flower development in *Liriodendron chinense*, a common ornamental tree species in southern China. Their findings were based on integrating small RNA, degradome, Illumina, and PacBio sequencing data [21]. Besides, the characterization and comprehensive analysis of lncRNAs involved in the flowering development of trifoliate orange (*Poncirus trifoliata* L. Raf.) was done by Wang et al. [22] through performing paired-end strand-specific RNA sequencing (ssRNA-Seq). Likewise, Shea et al. [23] identified long noncoding RNA in *Brassica rapa* L. following vernalization by performing similar methods of obtaining RNA-seq data.

Therefore, there is a critically important role of lncRNAs in controlling flowering time in plants that are known for bolting. Hence, to provide a novel insight into lncRNAs activity mechanisms in lettuce flowering, we identified flowering/bolting-related lncRNAs using transcriptome sequencing data in two lettuce lines with different bolting times. Besides, we performed a quantitative reverse transcription PCR (RT-qPCR) analysis to validate lncRNAs expression and constructed a co-expression network using weighted gene co-expression network analysis (WGCNA) based on differentially expressed mRNAs and lncRNAs. In summary, the flowering-associated function of identified lncRNAs was unveiled by predicting the potential lncRNA trans-regulated target genes involved in the six main flowering-related pathways. The potential novel lncRNA- miRNA- mRNA interactions in regulating the lettuce flowering/bolting process were also discovered. Additionally, the relationships between co-expressed lncRNAs and master regulators modulating the switch to flowering were unraveled within the identified key modules. The outcome of this research can be used as a baseline for subsequent future functional genomics studies on understanding the molecular basis underlying the lettuce flowering process.

## Results

### Identification of novel lncRNAs

**Fig. 1:**
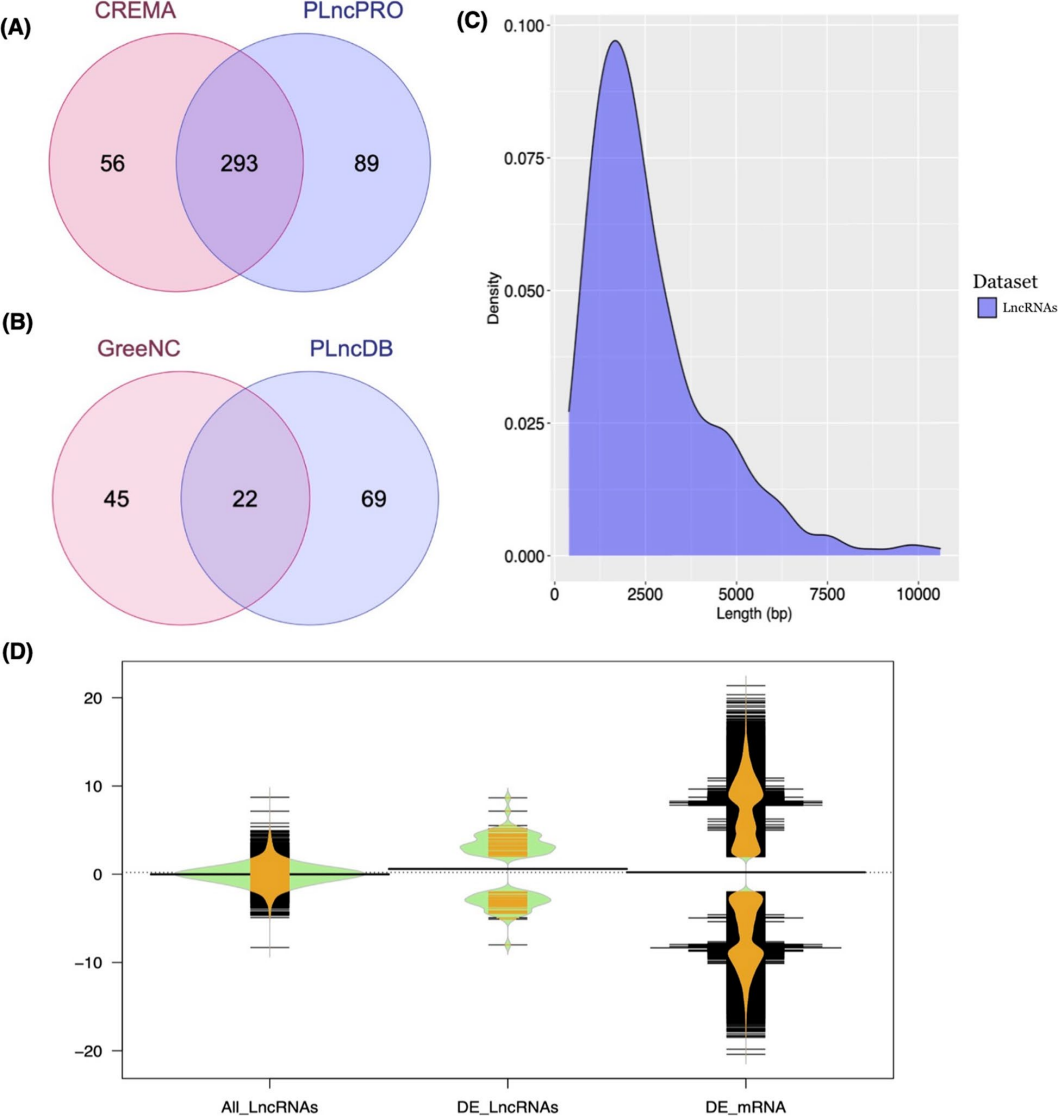
**A** Venn diagrams illustrating the number of both shared and distinct lncRNAs predicted by PLncPRO and CREMA. **B** Venn diagrams illustrating the BLAST results of lncRNA sequences against GreeNC and PLncDB databases. **C** Length distribution of lettuce lncRNAs. **D** Bean plots represent the differential expression levels of the lncRNA and mRNA transcripts. DE refers to differentially expressed genes

To identify the novel lncRNAs associated with flowering/bolting in *L. sativa*, two RNA-seq datasets under the BioProject accession numbers PRJNA325073 [9] and PRJNA422994 [3], with respectively 6 and 16 cDNA libraries, were exploited. These data belonged to line S24 as a bolting resistance line and S39 as a bolting sensitive line. Indeed, both lines S24 and S39 belong to leafy lettuce with serrated leaves. As compared to S24, line S39 generally grows faster and its leaves are bigger. More importantly, S39 is prone to bolting and is very sensitive to high temperatures, where S24 is bolting resistant and heat intensive. For this case, about 1121.1 million raw reads were analyzed utilizing a pipeline shown in Fig. S1. After quality control and trimming, high-quality clean reads (score > Q30) with a minimal length of 50 bp were aligned to the lettuce reference genome (*L. sativa* cv Salinas V8, https://genomevolution.org/coge/GenomeInfo.pl?gid=28333) using HISAT2 [24]. The percentage of mapped reads was between 78 and 98. Using StringTie v.2.1.1 [25], 186,558 transcripts were identified and quantitated. Among these, 97,041 transcripts in class codes of ‘u’ (intergenic), “o” (generic overlap with known exon), “i” (intronic), and “x” (overlap with a known gene on the opposite strand) were recognized as unannotated transcripts, of which 1114 transcripts were expressed with CPM > 1 in at least 21 samples. After filtering transcripts with lengths of > 200 bp and < 15 Kb, 924 transcripts remained. In the next step, 835 transcripts non-homologous to tRNA and rRNAs were screened. After excluding transcripts with at least one significant (E-value,1e-5) hit against the UniProt release, 512 remaining transcripts were assessed for protein-coding potential using the CPC2 [26] (https://github.com/gao-lab/CPC2_standalone). By using this program, 450 noncoding transcripts were detected, and inputted into PLncPRO [27] (https://github.com/urmi-21/PLncPRO), and CREMA [28] (https://github.com/gbgolding/crema) for rigorous prediction of novel lncRNAs. Ultimately, 293 potential lncRNAs (Table S1) were predicted by both PLncPRO and CREMA (Fig. 1A). In this study, 51 differentially expressed lncRNAs (DE-lncRNAs) were identified by the DESeq2 package on the IDEAMEX web server, using the FPKM value. The results of the filtration-based lncRNA identification procedure are summarized in Table 1.

The length distribution of identified lncRNAs showed that they are distributed from 393 nt to 10,600 nt (Fig. 1C). Compared to mRNA transcripts, lncRNAs were longer in length but fewer in exon number (Fig. S2). The log fold change (logFC) of all identified lncRNAs, DE-lncRNAs, and DE-mRNAs were also compared. The expression distribution results demonstrated that mRNAs had significantly higher expression diversity than lncRNAs. Additionally, the logFC of all lncRNA and DE-lncRNAs was differently distributed. The highest density of DE-lncRNAs was found with the approximate logFC range of − 2 to − 5 and 2 to 5, while a large number of lncRNAs appeared nearly with − 2 < logFC < 2 (Fig. 1D).

To obtain a deeper insight into the conservation of lncRNAs identified in this study, their sequences were aligned with plant lncRNA sequences publicly available in the GREENC [29], and PLncDB v.2.0 [30] databases. The BLAST search results demonstrated that 146 lncRNAs (~ 50% of all pulled-out lncRNAs) had at least one significant hit. Furthermore, around 24% of lncRNAs displayed significant homology with only lncRNA sequences in the GreeNC database, and 15.35% of candidate lncRNAs had significant hits solely in the PLncDB database (Fig. 1B).

**Table 1:** The results of filtration step in the lncRNA identification pipeline. Numbers represent the total number of transcripts filtered out in each step

Step	Number
Potential novel transcripts (Class codes: i, u, x, o, e)	97,041
Transcripts with CPM > 1 in at least 21 samples	1114
Transcripts with length > 200 bp and < 15 kb	924
Transcripts after filtering out tRNAs and rRNAs	835
Transcripts with no significant hit against UniProt, Pfam, and Rfam.	512
Noncoding transcripts predicted by CPC2	450
Novel LncRNAs predicted by PLncPRO, and CREMA	293

### Identification and functional enrichment analysis of potential target genes

To investigate the possible regulatory function of cis-acting lncRNAs, protein-coding genes from 100 kb upstream and downstream of known and novel lncRNAs were explored. In this regard, for 768 known lncRNAs, 1753 and 1544 potential cis-regulated target genes were deciphered upstream and downstream of lncRNAs, respectively. For 293 novel lncRNAs detected in this study, 1337 and 1159 upstream and downstream target genes were identified. Besides, 51 DE-lncRNAs were found to be adjacent to 231 upstream and 262 downstream target genes. Subsequently, to obtain a deeper understanding of the probable cis-regulatory function of both known and novel lncRNAs, GO enrichment and KEGG pathway enrichment analyses were conducted on the screened potential target genes.

**Fig. 2:**
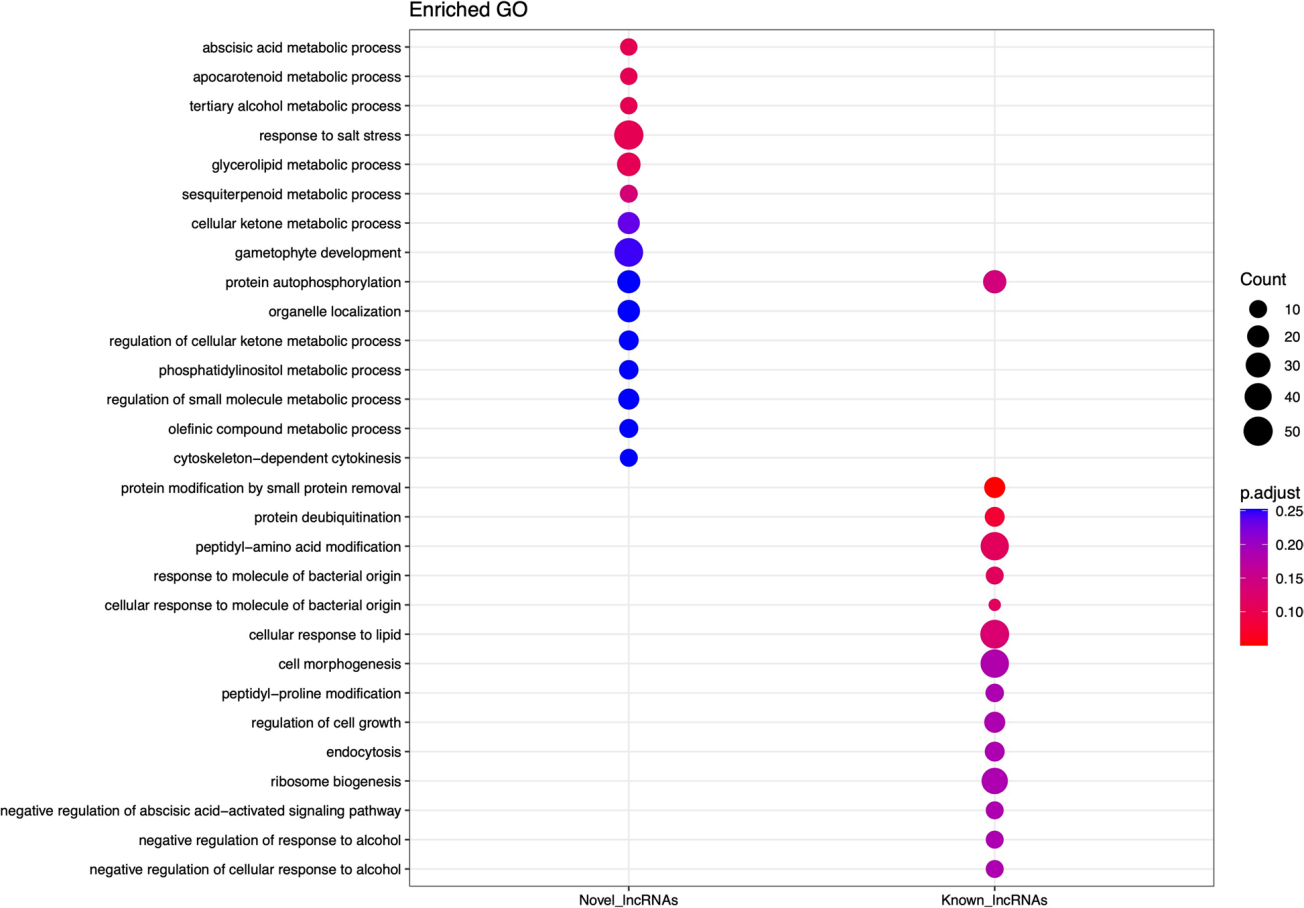
GO enrichment (BP: biological process) of protein-coding transcripts located 100 Kbp upstream and downstream of both known and novel lncRNAs

**Fig. 3:**
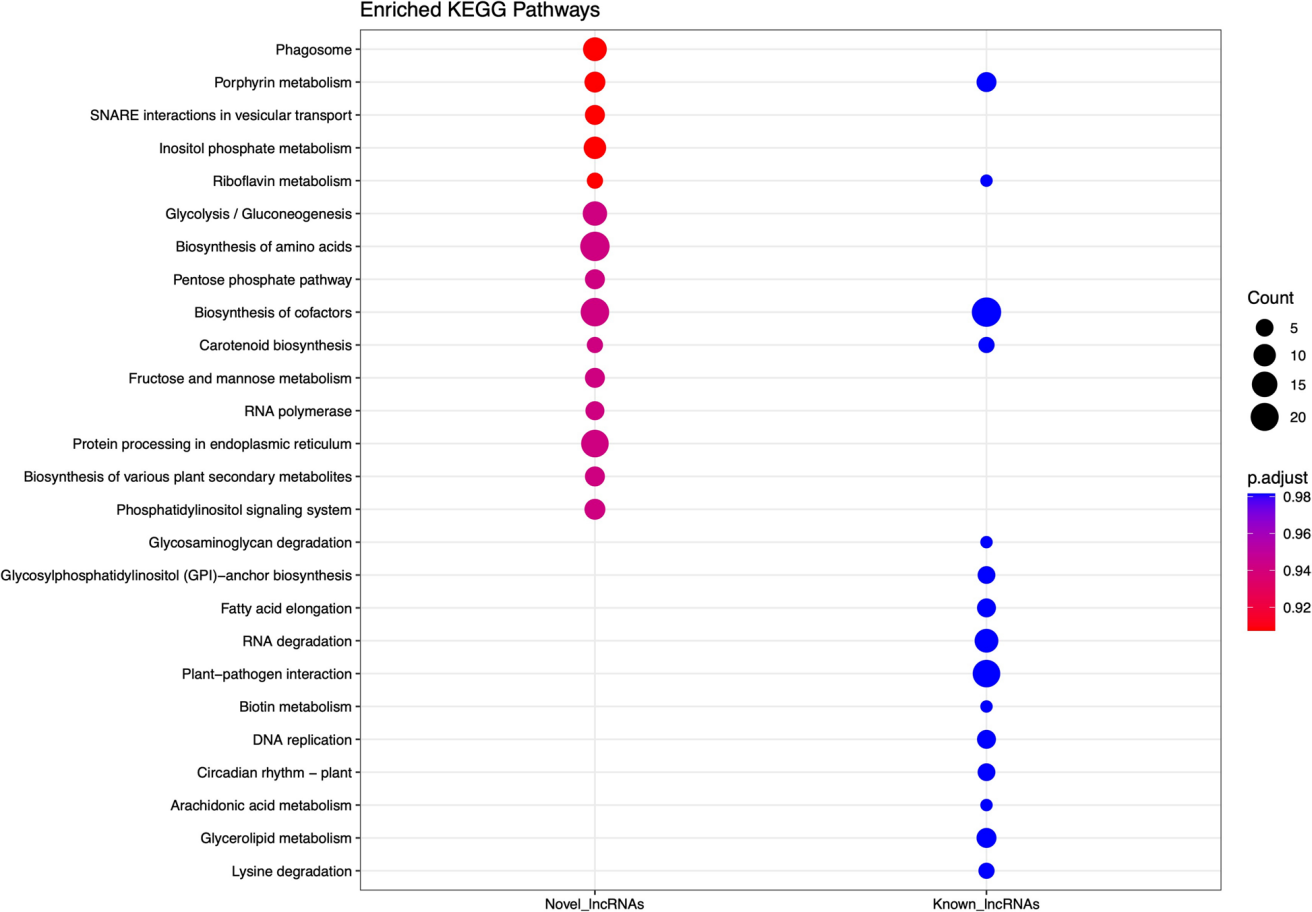
KEGG enrichment of protein-coding transcripts located 100 Kbp upstream and downstream of both known and novel lncRNAs

The GO enrichment analysis revealed that potential target genes of known lncRNAs and novel lncRNAs were enriched completely differently. Most known lncRNA cis-regulated potential target genes were mainly enriched into biological processes associated with “Peptidyl- amino acid modification”, “Cellular response to lipid”, and “Cell morphogenesis”, while for novel lncRNA potential target genes, the most highly enriched category was “Gametophyte development”, as a GO term directly associated with flower development (Fig. 2). The KEGG enrichment analysis results showed that “phagosome”, “Porphyrin metabolism”, “SNARE interactions in vesicular transport”, “Inositol phosphate metabolism”, and “Riboflavin metabolism” were significantly enriched among upstream and downstream novel lncRNAs target genes. The target genes of known lncRNA were mainly enriched in “biosynthesis of cofactors” and “plant-pathogen interaction”. Furthermore, enrichment analysis indicated that these genes were exclusively assigned to “circadian rhythm-plant”, as an indispensable pathway during the floral transition (Fig. 3).

### Assessment of lncRNAs-mRNAs interaction using LncTar

**Fig. 4:**
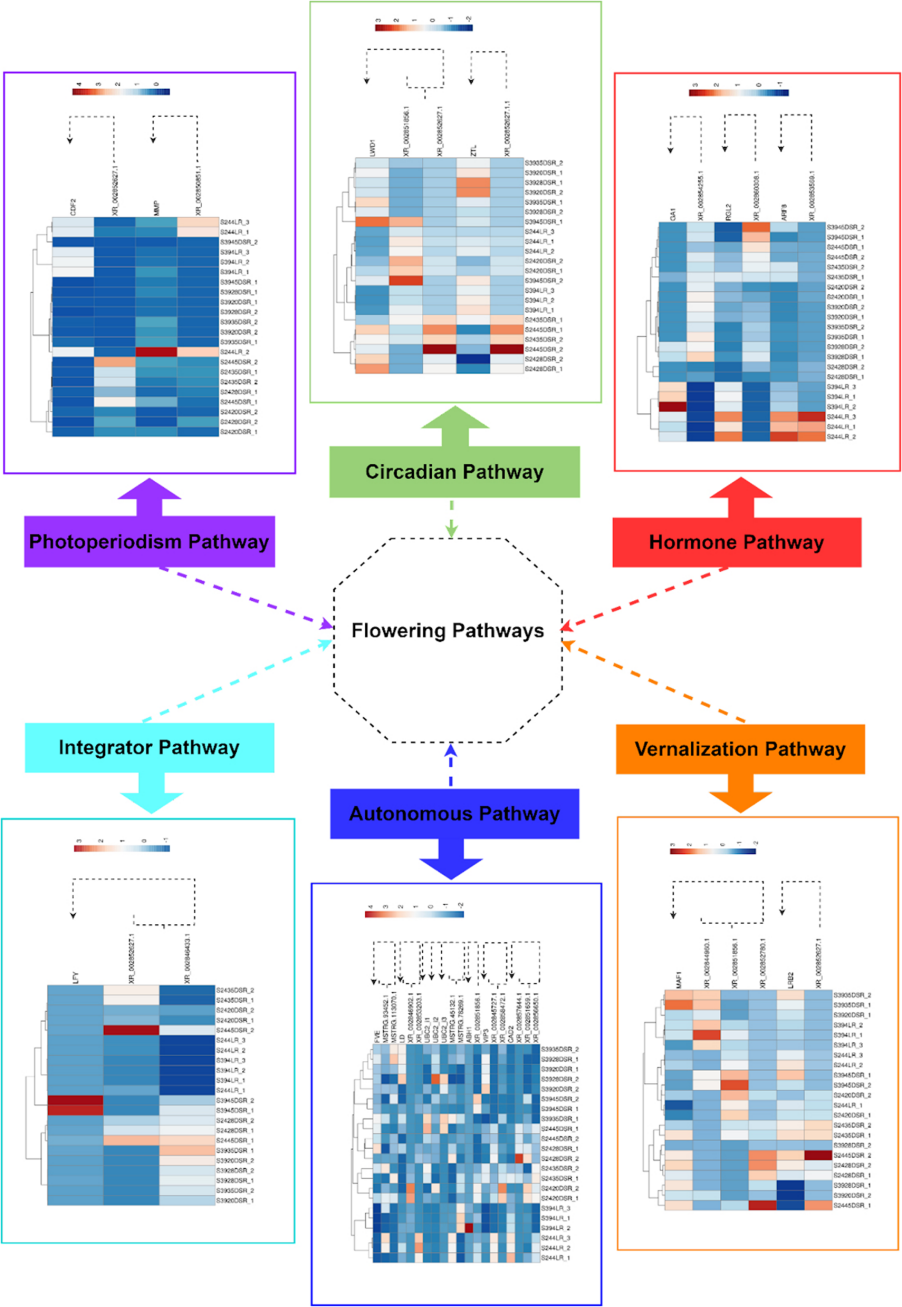
Flowering-related pathways unraveled through the prediction of potential lncRNA trans-regulated target genes by lncTar

Since lncRNA-mediated regulation is also carried out by base-pairing with complementary mRNAs [13], LncTar (http://www.cuilab.cn/lnctar) was employed to predict potential lncRNA trans-regulated target genes. Interestingly, several predicted target genes were found to be involved in flowering-related pathways. According to the results, two DE-lncRNAs including, XR_002852627.1 and XR_002850851.1 were identified to target XM_023917191.1 (Dof-type zinc finger domain-containing protein; *CDF2*) and XM_023876432.1 (Matrix metalloprotease; *MMP*), respectively, which are involved in photoperiodism pathway. The circadian pathway-associated target gene, XM_023884739.1 (LIGHT-REGULATED WD 1; *LWD1*) was shown to be potentially regulated by DE-lncRNAs XR_002851856.1, and XR_002852627.1. Another circadian target gene was XM_023909739.1 (ZEITLUPE; *ZTL*), which was detected as a trans-target of XR_002852627.1. The participation of two DEGs, XM_023886038.1 (MADS AFFECTING FLOWERING 1; *MAF1*) and XM_023890738.1 (LIGHT-RESPONSE BTB 2; *LRB2*), has been asserted in the vernalization pathway. In this study, *MAF1* was found to be regulated by three DE-LncRNA including, XR_002844960.1, XR_002851856.1, and XR_002852780.1. Furthermore, LRB2 was detected as the target of XR_002852627.1. LEAFY (XM_023888266.1; LFY), a gene involved in the integrator pathway, was predicted to be the target gene of XR_002852627.1 and XR_002846433.1. The trans-regulatory activity of XR_002854255.1 on XM_023882685.1 (GA REQUIRING 1; *GA1*), XR_002860308.1 on XM_023879739.1 (REPRESSOR OF GA; *RGA*), and also XR_002853569.1 on XM_023891863.1 (AUXIN RESPONSE FACTOR 8; *ARF8*) was also predicted by lncTar. The involvement of these flowering-associated target genes was detected in the hormone pathway. Another flower-related pathway identified in this study was “autonomous” in which XM_023902615.1 (*FVE*), XM_023892071.1 (LUMINIDEPENDENS; *LD*), XM_023917173.1 (UBIQUITIN-CONJUGATING ENZYME 2; *UBC2_i1*), XM_023917174.1 (*UBC2_i2*), XM_023917175.1 (*UBC2_i3*), XM_023881249.1 (ABA HYPERSENSITIVE 1; *ABH1*), XM_023901095.1 (VERNALIZATION INDEPENDENCE 3; *VIP3*), and XM_023905511.1 (CADMIUM SENSITIVE 2; *CAD2*) were revealed to be targeted by both novel and known DE-lncRNAs (Fig. 4).

### Assessment of lncRNAs as the competing endogenous target mimics (eTMs) for miRNAs

In the current study, lncRNAs were also investigated for the possibility of being miRNAs’ eTMs using the psMimic algorithm [31]. According to the results, a total of 17 novel lncRNAs (Table S2) were predicted to be potential eTMs for 15 miRNAs. Of these, six lncRNA were found to be DE-lncRNAs. In further analysis, utilizing the psRobot toolbox [32], 695 putative unique target genes were identified for 15 miRNAs that previously exhibited mimicry with lncRNAs. Among predicted target genes, the putative flowering-related genes including, protein CDC73 homolog (*PHP*) involved in the vernalization pathway, cyclic dof factor 1-like (*CDF2*), metalloendoproteinase 3-MMP-like (*At2-MMP*) involved in photoperiodic pathway, lysine-specific histone (*LDL2*), protein MODIFIER OF SNC1 1 (*MOS1*), pre-mRNA-processing-splicing factor 8 A (*PRP8*), and protein MRG1 (*MRG1*) involved in autonomous pathway were detected.

Similarly, among known lncRNAs, a total of 18 DE_lncRNAs were predicted to be potential eTMs for 18 miRNAs (Table S3), of which 5 miRNAs were shared with novel lncRNAs (Lsa-miRN1644, Lsa-miRN1673, Lsa-miRN1704, Lsa-miRN1716, Lsa-miRN1719). Additionally, 782 putative unique target genes for 18 miRNAs were predicted. Among predicted target genes, *PHP* involved in the vernalization pathway, *CDF2* and light-mediated development protein DET1 (*DET1*) involved in photoperiodism, *LDL2*, *MOS1*, *PRP8*, LOW QUALITY PROTEIN: homeobox protein LUMINIDEPENDENS (*LD*) and histone-lysine N-methyltransferase ATXR7 isoform X1 (*ATXR7*) involved in autonomous, and two-component response regulator-like APRR3 (*PRR3*) involved in circadian clock were found as the putative flowering-related genes.

### Co-expression network analysis

To acquire a general view on the regulatory networks involved in the lettuce flowering process, WGCNA analysis was conducted to construct a gene co-expression network of the mRNAs and lncRNAs. Utilizing the hierarchical clustering of 1-TOM and the DynamicTree Cut algorithm, 4,539 unique DEGs, 51 novel DE-lncRNAs, and 768 known DE-lncRNAs were categorized into 8 modules with distinguished colors (Table S4).

The blue module contained the largest number of DE-lncRNAs (225 known DE-lncRNAs and 19 novel DE-lncRNAs). On the other hand, the salmon module, with 23 known DE-lncRNAs and 1 novel DE-lncRNAs, included the lowest number of DE-lncRNAs.

Regarding WGCNA analysis, in each module (except for the greenyellow), the novel relevance between DE-lncRNAs and flowering-associated genes were deciphered, which can reveal the lncRNA/mRNA interaction mediated regulation of the flowering process. For instance, in the blue module, the potential association was detected between lncRNAs and 26 neighboring DEGs involved in the regulation of flowering. These differentially expressed genes include EARLY FLOWERING 9 (*ELF9*), *FVE*, CONSTANS (*CO*), RED AND FAR-RED INSENSITIVE 2 (*RFI2*), GA REQUIRING 2 (*GA2*), GIBBERELLIC ACID INSENSITIVE (*GAI*), PHOSPHOGLUCOMUTASE, STARCH-FREE 1 (*PGM*), PSEUDO-RESPONSE REGULATOR 9 (*PRR9*), PHYTOCHROME E (*PHYE*), INSENSITIVE DWARF1B (*GID1B*), and ABRE BINDING FACTOR 4 (*ABF4*). The possible relationship between DE-lncRNAs and 11 flowering-related genes such as MADS AFFECTING FLOWERING 1 (*MAF1*), TERMINAL FLOWER 1 (*TFL2*), MEDIATOR 18 (*MED18*), BHLH48, HISTONE H2A PROTEIN 9 (*HTA9*), TBP-ASSOCIATED FACTOR 15B (*TAF15B*), and MULTICOPY SUPRESSOR OF IRA1 (*MSI1*) were unraveled in the magenta module. The co-expression of DE-lncRNAs with TILTED 1 (*TIL1*), SQUAMOSA promoter-binding protein-like 10 (*SPL10*), and LUMINIDEPENDENS (*LD*), as the DEGs involved in flowering regulation was found in the pink module. In the rest of the modules, DEGs included FLOWERING LOCUS C EXPRESSOR-LIKE 4 (*FLL4*) (in the purple module), gibberellin 3-beta-dioxygenase 1-like and BTB/POZ domain-containing protein POB1-like (in the salmon module), and COLD, CIRCADIAN RHYTHM, AND RNA BINDING 2 (*CCR2*) (in the tan module). These DEGs were the lncRNA’s neighboring flowering-associated genes. To evaluate the relationship among co-expression modules and days after planting traits, the Pearson correlation coefficient analysis was performed between eigengene values in each module and the individual gene expression values related to different growth stages in S24 and S39 lines. As shown in the module-trait relationship heatmap (Fig. S3), the significantly correlated modules exhibited low and mostly positive correlation coefficient values. These significant positive correlations were observed in the modules, including blue (in S39_4L), magenta (in S24_20DS), pink (in S24_45DS), salmon (in S24_4L), purple (in S39_20DS). Furthermore, the greenyellow and tan modules were positively correlated with S39-35DS (concurrent with bolting time in S39), while conversely, a negative correlation was observed between these modules and S24-35DS. These opposite correlation coefficient values can represent the eigengenes in these modules and/that may regulate early bolting in S39. Besides, the purple module was positively correlated with S39_20DS, while negatively correlated with S39-35DS. This observation suggested that genes in this module may negatively regulate bolting in S39.

### LncRNA expression study by qPCR

**Fig. 5:**
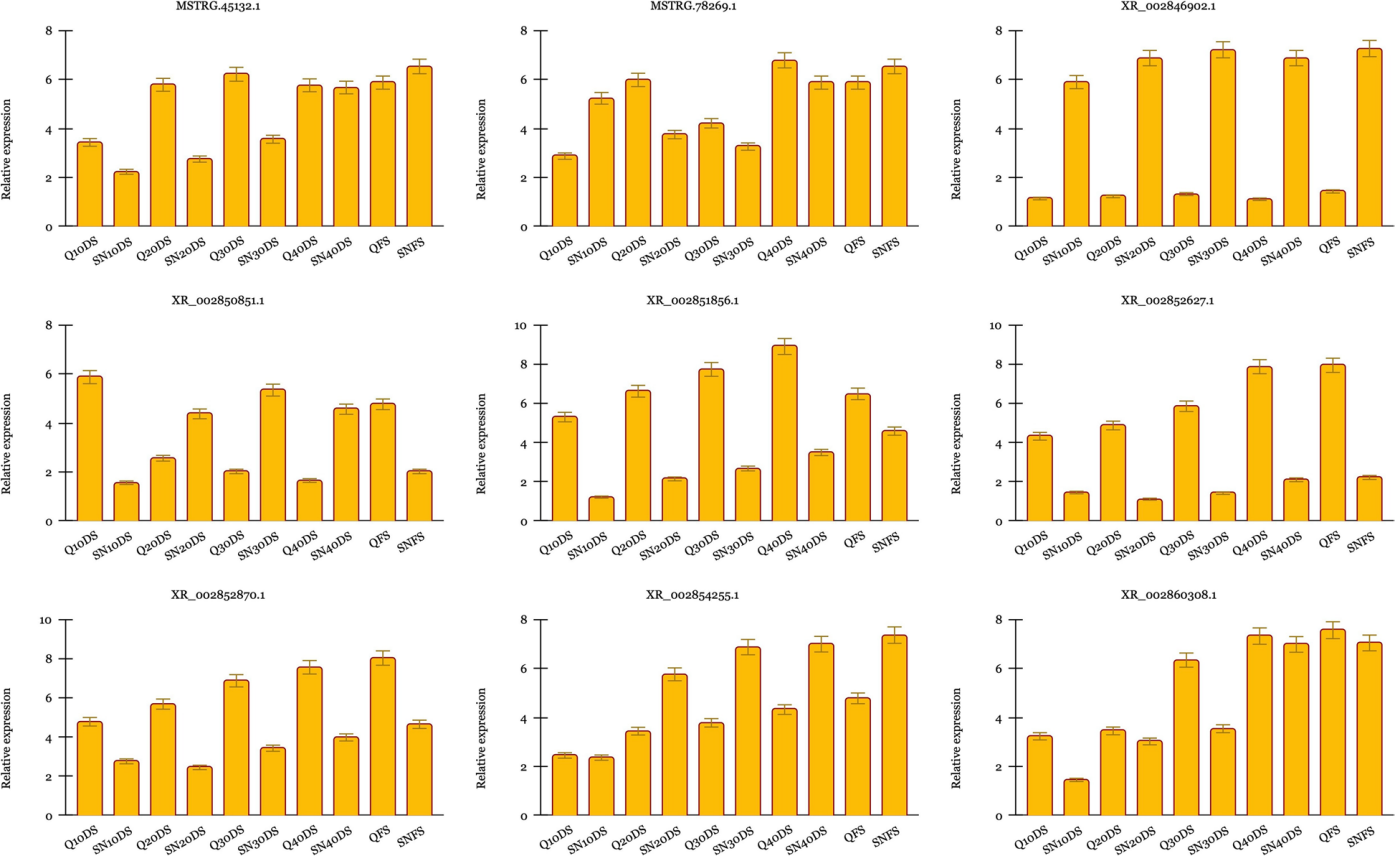
Relative expression of nine selected lncRNAs by qPCR collected from shoot apical meristem samples in 5 different growth stages in two different lettuce genotypes, Qomi (early bolting) and Sefid-e-Neishabour (late bolting). Q, SN, D, S, and F represent Qomi, Sefid-e-Neishabour, days after planting, flowering and shoot apical meristem, respectively. The examined days after planting are included; 10, 20, 30, and 40. Bars display mean values of three replicates (+/− standard deviation). *p-value < 0.05 and **p-value < 0.01 indicate significant differences

To accredit the expression of predicted DE-lncRNAs, qPCR analysis was performed for nine DE-lncRNAs in two different lettuce genotypes, Qomi and Sefid-e-Neishabour, which were grouped by [33] as the early and late bolting genotypes, respectively. A near concordance between the expression results obtained from qPCR and RNA-Seq was observed. According to the qPCR expression data obtained from shoot apical meristem samples in 5 different growth stages (from vegetative to reproductive growth stage), it was demonstrated that the expression of XR_002846902.1 and XR_002854255.1 were significantly up-regulated in the late bolting genotype compared to the early bolting one. On the other hand, XR_002851856.1, XR_002852627.1, XR_002852780.1, and XR_002860308.1 were found to indicate a higher expression in the early bolting compared to the late one (Fig. 5). Concerning both the RNA-seq and qPCR results, the upregulation of XR_002850851.1 was observed during different vegetative growth stages in both late bolting lettuce plants, Sefid-e-Neishabour (evaluated by qPCR) and S24 (evaluated by RNA-seq). The higher expression of this lncRNA in two late bolting plants could reveal its probable role in resistance against bolting followed by the delay in flowering time. On the other hand, MSTRG.45132.1 was detected with the highest expression in the early flowering stage Qomi and S39 (early bolting plants) in comparison with the late bolting ones. Hence, it can be inferred that this lncRNA can play a crucial role in inducing flowering.

**Fig. 6:**
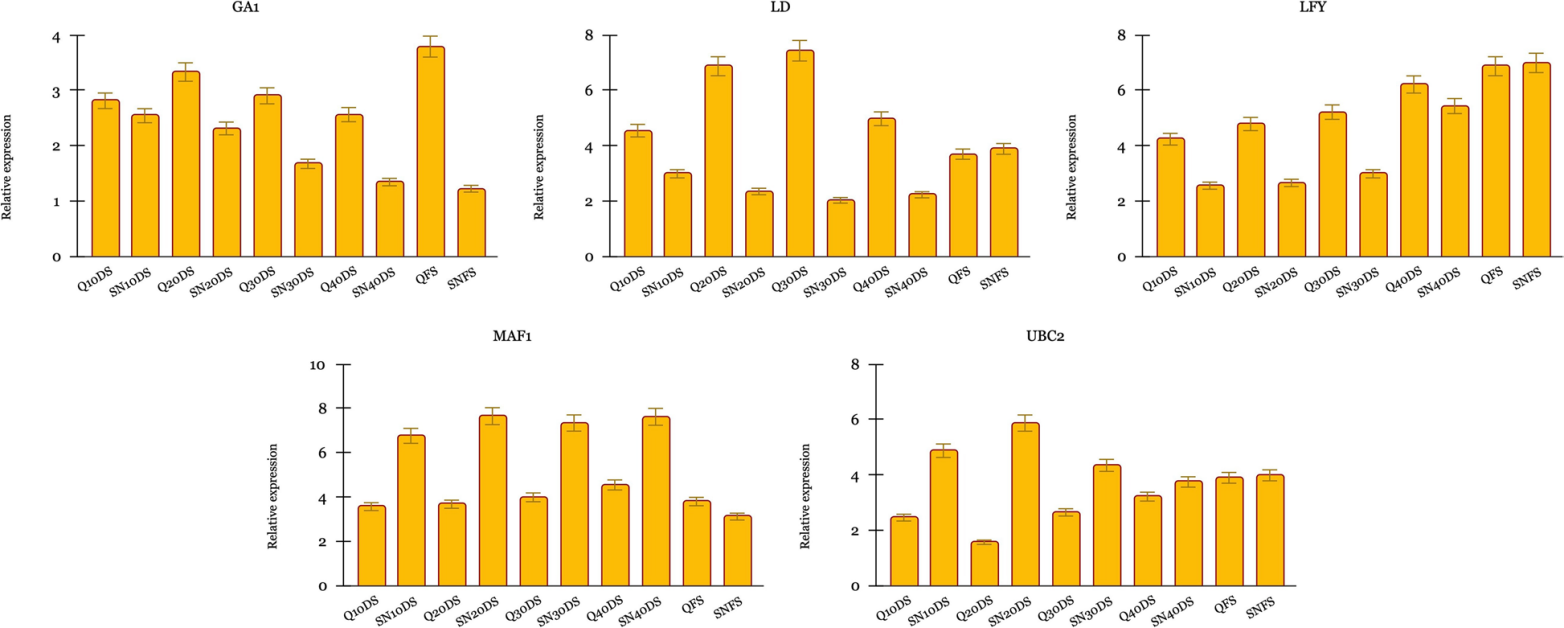
The relative expression of putative target genes determined by RT-qPCR in two genotypes at different development stages. *p-value < 0.05 and **p-value < 0.01 indicate significant differences

In further, we evaluated the expression of target genes of bolting/flowering-related lncRNAs by RT-qPCR (Fig. 6). These results revealed that the selected coding genes (target genes) expression patterns exhibited an inverse correlation with their corresponding lncRNAs, indicating that these lncRNAs could negatively regulate their corresponding target genes. *GA1*, *LD*, and *LFY* target genes corresponding to XR_002854255.1, XR_002846902.1, and XR_002852627.1 lncRNAs respectively indicated higher expression in vegetative stages of early bolting Qomi genotype. However, *UBC2* and *MAF1* associated with MSTRG.45132.1 and XR_002851856.1 lncRNAs, indicated higher expression in late bolting Sefid-e-Neishabour genotype.

## Discussion

In commercial lettuce varieties, premature bolting has a major negative impact on the quality and marketability through the thickening of the leaves and the induction of secondary metabolite production, which renders a bitter and disagreeable taste in the leaves [34]. Since flowering initiation is a highly intricate developmental patterning process influenced by the interconnections between environmental stimuli and molecular signaling pathways, broadening knowledge and understanding of the molecular basis underlying the lettuce flowering process is vital to restraining early bolting [3, 35]. LncRNAs are one of the major endogenous genetic components contributing to flowering time and flower development. They act as interplay with DNA, RNA and protein molecules, and they modulate the expression level of their targets at the epigenetic, transcriptional, post-transcriptional, or translational levels [36–38]. Until recently, several lncRNAs have been revealed to implicate a major role in the flowering regulation of some model plant species, particularly *Arabidopsis thaliana* [39]. Over the past decade, different genetic and molecular studies have been performed to unravel the putative genes and pathways controlling the flowering/bolting process in lettuce [7].However, the knowledge about the pivotal role of lncRNAs, particularly in the transition from the vegetative to reproductive phase as well as the flowering time, is still enigmatic. Accessing lncRNAs and identification of the lncRNA-mRNA co-expression networks can also provide a holistic insight into the architecture of gene regulatory networks regulating flower development in lettuce. Hence, in this study, the deep transcriptome datasets obtained from two early and late-bolting near-isogenic lettuce lines in different developmental stages from vegetative to reproductive phase were analyzed for the identification and characterization of the potential flowering/bolting-associated lncRNAs. The framework of this study was briefly summarized in the following steps: identification of novel lncRNAs, evaluation of lncRNAs conservation, differential expression analysis of mRNAs and lncRNAs, prediction of the potential function of lncRNAs, validation of lncRNAs expression by qPCR, and construction of the regulatory lncRNA-mRNA co-expression network using the WGCNA analysis.

Utilizing a rigorous multi-filter bioinformatic pipeline, 293 novel lncRNAs were identified, 51 of which were DE-lncRNAs. To precisely clarify the lncRNAs-mediated regulatory mechanisms, GO enrichment and KEGG pathway analysis were conducted on the cis-acting target genes of DE-lncRNAs (both known and novel lncRNAs). GO enrichment results displayed that cis target genes of novel and known lncRNAs were enriched in 29 and 15 terms, respectively, among which, “Gametophyte development” is directly associated with the flowering process [40, 41].

Indeed, the enrichment of cis-acting target genes of lncRNAs in “Gametophyte development” suggests the probable function of lncRNAs in flower development by modulating genes associated with gametophyte production. Several studies have also unveiled the major roles of ncRNAs in governing processes involved in the development of haploid male and female gametophytes [42–45]. Based on KEEG analysis, it was found that the cis-acting target genes of both known and novel lncRNAs were enriched in a total of 26 pathways. Among these pathways,, the “Circadian rhythm-plant” has been recognized as a core pathway in the regulation of flowering time [46]. To ensure reproductive success, precise flowering time regulation is essential. Hence, plants require an endogenous timekeeper to keep track of the daylight changes, as a prominent determinant for onset of flowering [47]. Until now, the array of circadian clock-related components has been unraveled, which can synchronize molecular and physiological processes to the exogenous day/night cycles (light signaling) [47–49]. However, the KEEG results of this study demonstrated that the part of lncRNAs probably significantly regulates the floral transition time by regulating circadian clock-associated genes within the “Circadian rhythm-plant” pathway.

To help deepen our understanding of lncRNAs function, trans-regulatory target genes of lncRNAs were also predicted, and the remarkable novel associations were deciphered between DE-lncRNAs (known and novel) and target genes involved in six flowering-related pathways including autonomous, integrator, vernalization, hormone, circadian, and photoperiodism pathways. The overall assessment demonstrated that trans-acting lncRNAs in lettuce probably have more participation in the flowering process than those acting in cis mode. In the autonomous pathway, a relationship was revealed between two novel lncRNAs (MSTRG.93452.1 and MSTRG.113070.1) and the *FVE* gene. The *FVE/MSI4* gene has a positive effect on flowering time regulation [50]. During the autonomous pathway, *FVE* contributes with two histone deacetylase HDA6 and the histone demethylase FLD and forms a multiprotein complex to repress *FLC* expression via histone modifications in *FLC* locus (decrement of H3K4 trimethylation and H3 acetylation) [51, 52]. In our study, identification of the novel regulatory interaction between the *FVE* gene and pertinent novel lncRNAs suggests that the lncRNAs-mediated epigenetic regulation of *FLC* is followed by flowering initiation via potentially targeting the *FVE* gene. Another target gene belonging to the autonomous pathway was *LD*, which was predicted to be targeted by XR-002846902.1 and XR-002853203.1. The positive regulatory role of this gene in promoting the floral transition was displayed by Lee et al. [53]. Additionally, this gene has been found with the most conspicuous effect on circadian periods [54]. Besides, Aukerman et al. [55] unveiled the *LD*- mediated *LFY* modulation. *LFY* is known as the key transcription factor in the flower formation process during the earliest floral stage [56], and also a pivotal integrator in modulating the floral gene network [57]. Concerning the interaction between *LD* and *LFY*, we can confer the regulatory role of XR-002846902.1 and XR-002853203.1 on *LFY* gene, and thus the floral meristem transition via targeting *LD*. Three *UBC2* isoforms (1–3) were the other autonomous-related genes that were predicted as the trans targets of MSTRG-45132.1 and MSTRG.78269.1. The *UBC2* has been found as the positive regulator of *FLC* and repressor of flowering [58, 59]. In the model presented by Cao et al. [60], a heterotetramer consisting of two HUB1 (HISTONE MONOUBIQUITINATION1) and two HUB2 interacts with UBC1/2 to monoubiquitinate H2B located in the *FLC* chromatin. Followed by this modification, the histone H3 methyltransferase is employed to induce H3K4 and H3K36 hypermethylation, and ultimately, *FLC* upregulation. In the current study, our findings suggest that MSTRG-45132.1/MSTRG.78269.1 probably mediated epigenetic regulation of *FLC* chromatin by modulating the UBC1,2/HUB1,2. Another lncRNA/mRNA interplay-mediated flowering regulation within the autonomous pathway was found between *ABH1* and XR-002851856.1. The negative role of *ABH1* in flowering has been proved via modulating three major floral regulators including *CO*, *FLC* and *FLM* [61, 62]. Our study displayed the potential regulatory role of two previously predicted lncRNAs, XR-002845727.1 and XR-002858472.1 on *VIP3*. The negative role of *VIP3* was initially revealed in regulating the flowering time via activating the *FLC* expression [63]. As a putative subunit of the RNA polymerase II-associated complex (PAF1c), *VIP3* participates in *FLC* up-regulation by induction of methylation of histones H3K4 within *FLC* chromatin [64, 65]. Therefore, the activity of *FLC* is probably modulated via potential trans-regulation mediated by XR-002845727.1 and XR-002858472.1 on *VIP3*. Additionally, a collaborative-based association was unraveled between *CAD2*, a gene located in the autonomous pathway, and its relevant lncRNAs including XR-002857644.1, XR-002851659.1, and XR-002856650.1. The positive regulatory role of *CAD2* on flowering time has been demonstrated by Ogawa et al. [66]. The *CAD2* gene is involved in biosynthesis of the glutathione (GSH) [67] which is necessitated for floral meristem initiation [68, 69], and its accumulation directly affects flowering time [70]. In the integrator pathway, the trans-regulatory role of two lncRNAs, XR-002852627 and XR-002846433.1, was revealed on the *LFY* gene. The members of the LFY family have been known as the floral meristem identity genes [71] and function as the decisive integrators in modulating the floral gene network [57]. For example, LFY TF promotes the expression of the master floral regulator, *APETALA1* (*AP1*), by mediating AP1 locus activation via translocating linker histone H1, and recruiting SWI/SNF chromatin remodelers locus [72]. Hence, lncRNA-mediated modulation of *LFY* expression by XR-002852627 and XR-002846433.1 can unveil the novel components in regulating this pioneer TF and other downstream floral regulators. The probable regulatory role of lncRNAs in the vernalization pathway was found through predicted trans-regulation of XR-002844960.1, XR-002851856.1, and XR-002852780.1 on *MAF1/ FLM*. In comparison with flowering-related genes which are also involved in other plant growth aspects, *MAF1* has been seemingly found to act only on flowering time regulation [73]. This gene, encoding a MADS-box TF associated with *FLC*, functions as a repressor of flowering during vernalization [74, 75]. In fact, the ratio of two splice variants, FLM-β and δ, which are affected by temperature fluctuations (between 16 and 27 °C) determines the function of *FLM* in fine-tuning of flowering time [75]. The second novel collaborative-based relationship in the vernalization pathway was found between *LRB2* and XR002852627.1. The positive regulatory role of *LRB2* in flowering time was deciphered by Christians et al. [76]. The LRBs regulate floral induction through impacting the degradation of FRIGIDA (*FRI*), which regulates vernalization and timing of transition from the vegetative to reproductive phase [77]. Followed by degradation of *FRI*, there is an overexpression of lncRNA COOLAIR and then a reduction in the level of histone H3Lys4 trimethylation (H3K4me3) in *FLC* chromatin. The sum of these changes leads to promoting flowering [78]. The potential trans-regulatory role of lncRNAs in lettuce flowering was also revealed within the hormone pathway. For instance, XR-002854255.1 was predicted to regulate *GA1*. This gene is located in the early stage of the GA biosynthesis pathway and positively regulates flower formation through coordination and interaction with *LFY* [79, 80]. Thus mutations in *GA1* result in failure or delay in flower induction [79]. Furthermore, a potential association was uncovered between XR-002860308.1 and *RGL2*, as another flowering-related target gene in the hormone pathway. The product of *RGL2*, as a DELLA protein and negative modulator of GA response, is vital for repressing flowering [81, 82]. This gene, like other *DELLA* genes, exerts its function chiefly by interacting with *FLC* and *SPL* to block floral transition [83, 84]. Interaction between *ARF8* and XR-002853569.1 was another predicted lncRNA/mRNA interaction within the hormone pathway. The *ARF8* gene is involved in promoting jasmonic acid production and flower maturation via repressing the Class 1 KNOX genes, as major inhibitors of floral organs’ elongation and differentiation [85–87]. In looking for a novel result, we deciphered a novel crosstalk between miR167, *ARF8*, and XR-002853569.1 candidates for mediating the maturation of lettuce floral organs.

Within the circadian rhythm pathway, we detected the plausible trans-regulatory role of XR-002851856.1 on *LWD1*. *LWD1* acts on photoperiodic flowering regulation and period length through modulating the expression of multiple oscillator genes including CIRCADIAN CLOCK ASSOCIATED1 (*CCA1*) and PSEUDO-RESPONSE REGULATOR9 (*PPR9*) [88, 89]. The other novel lncRNA/mRNA interaction in the circadian rhythm pathway was predicted between *ZTL*, an F-box protein, and XR-002852627.1. *ZTL* has been revealed to negatively regulate the photoperiodic control of flowering time through reduction in the *CO* and *FT* transcript levels [90, 91]. In the photoperiodism pathway, a probable interaction was unraveled between *CDF2* and XR-002852627.1. Interestingly, it was found that XR-002852627.1 is engaged in coregulating both *ZTL* and *CDF2* within two detached pathways. In 2009, *CDF2* was first reported to be involved in delaying flowering by suppressing *CO* transcription [92]. Then, Sun et al. [93] revealed that *CDF2* could control the flowering time, seemingly independent of *CO* via miR172 suppression and miR156 activation during (DICER-LIKE 1) DCL1-mediated pri-miRNAs processing. Regarding *CDF2* function and discovering the lncRNA- mediated CDF2 trans-regulatory, our results suggest a new interplay between XR-002852627.1, *CDF2*, and miR156/miR172 in modulating flowering time within the photoperiodism pathway in lettuce. The final predicted the presence of lncRNA- governed trans-regulatory relationship between *MMP* and XR-002850851.1. Even though the positive relevance of *MMP* to flowering was demonstrated by Golldack et al. [94], the actual function of this MMP protease in modulating flowering is largely unknown. Hence, unraveling this novel relationship between *MMP* and XR-002850851.1 can provide a better understanding of characterizing the function of this gene in the flowering process.

In a deeper investigation into the diagnosis of lncRNAs’ function, our findings unraveled the potential novel lncRNA- miRNA- mRNA interactions in modulating the lettuce flowering process. It was found that XR-002852074, MSTRG.80963.1, and MSTRG.31850.1 all three together act as eTMs for Lsa-miRN1644 aiming to regulate *PHP* (involved in the vernalization pathway), and *CDF2* (involved in photoperiodic pathway). Both *PHP* and *CDF2* have been identified with a negative regulatory role in flowering. The *PHP*, a component of the PAF1 complex, is involved in modulating the expression of H3K27ME3-enriched genes, especially the major flowering suppressor, *FLC* [95]. The *CDF2*, a member of the DOF family, has been recognized as the delayed flowering factor by repressing *CO* transcription [92, 96]. In another novel interplay, XR-00284767.1 was found to act as eTM for Lsa-miRN1711 to regulate *DET1* (involved in photoperiodism pathway). According to findings of Kang et al. [97], *DET1* was detected to function in floral repression via epigenetically inducing *FLC* expression, and repressing *SOC1* through interacting with MSI4/FVE or preventing GIGANTEA (*GI*) from binding to the *FT* promoter, resulting in *FT* repression. Our results have also indicated that both XR-002859499.1 and MSTRG.126339.1 potentially function as eTMs for Lsa-miRN1704, aiming to modulate three genes participating in the autonomous pathway, including *LDL2*, *MOS1*, and *PRP8*. *LDL2* was found to act with FLOWERING LOCUS D (*FLD*) to prevent *FLC* expression by mediating histone H3 lysine 4 (H3K4) demethylation; thus, it provokes the floral transition [98, 99]. Similar to *LDL1*, *MOS1* was found to promote flowering. Bao et al. [100] demonstrated that *MOS1* negatively regulates the expression of *FLC* via inhibiting *SUF4*, a transcriptional activator of the *FLC*. PRP8 has been documented as a central component of the spliceosome [101]. In the study by Marquardt et al. [102], the PRP8-dependent suppression of *FLC* transcription by affecting the lncRNA COOLAIR splicing was discovered. Hence, the indirect effect of XR-002859499.1 and MSTRG.126339.1 on *FLC* can be inferred. From our results, XR-002851210.1- Lsa-miRN1726 was another novel potential regulatory component in regulation of flowering through modulating *LD* (involved in autonomous pathway). It was also predicted that XR-002845460.1 could act as a decoy for Lsa-miRN4414 to control the expression of *ATXR7*, as a Set1- class H3K4 methylase involved in *FLC* activation and proper expression via full H3K4 methylation [103]. XR-002847497.1 was another lncRNA which was found to act as a potential eTM for Lsa-miRN1728 to regulate *PRR3*. *PRR3*, as a negative regulator, controls the photoperiodic flowering response within the circadian clock pathway [104]. The MSTRG.111752.1- Lsa-miR1725 interplay, which mediated the potential post-translational regulation of *At2-MMP* (involved in the photoperiodic pathway), and *MRG1* (involved in the autonomous pathway) was also observed in our study. *MRG1*, a positively novel type of chromatin modulator, participates in photoperiodic regulation of flowering time [105, 106]. This gene, along with *MRG2*, function as the H3K4me3/H3K36me3 readers and activate the expression of *FT* via physically interacting with *CO* [105].

The construction of lncRNA-mRNA co-expression networks utilizing WGCNA could also corroborate the roles of the lncRNA-mRNA regulatory connections in the lettuce flowering process. Interestingly, relationships between co-expressed lncRNAs and leading protein-coding genes were more predominantly deciphered in the blue and magenta modules, which positively intervene the switch to flowering. These relationships and genes included *FVE* (in the blue module) and *TAF15b* (in the magenta module) via repressing *FLC* expression [51, 52, 107, 108], *PRR9* (in the blue module) via activating the *CO* and repressing CYCLING DOF FACTOR1 (*CDF1*) [109], *PGM* (in the blue module) via mobilizing carbohydrate reserves [110, 111], *MSI1* (in the magenta module) via affecting the floral integrator genes including, *CO*, *FT*, and *SOC1* [112, 113], and also *MED18* (In the magenta module) via mediating the floral meristem identity gene, AGAMOUS (*AG*), and *FLC* [114]. Besides, the key TFs involved in promoting flowering through modulating the major floral integrators, including *CO* and *ABF4* as well as *BHLH48*, were detected in these modules [115–119]. On the other hand, several flowering-related genes were found to contribute to the negative regulation of flower development including *ELF9* (in the blue module) via repressing *SOC1* [120], *RFI2* (in the blue module) via negatively regulating *CO* expression and photoperiodic flowering [121], *TFL2* (in the magenta module) via maintaining the elevated levels of H3K9 dimethylation at FLC chromatin [122, 123]. Identifying these key modules containing the remarkable numbers of significant flowering-associated genes could screen out the novel lncRNAs in these modules that presumably contribute to the same flowering-related functions that their co-expressed mRNAs are involved in.

### Conclusion

This study aimed to identify and characterize the potential flowering/bolting-associated lncRNAs in lettuce by employing RNA-seq data. The pivotal roles of lncRNAs in transition from the vegetative to reproductive phase were unraveled through GO enrichment and KEGG pathway analysis conducted on the lncRNAs cis-acting target genes, identifying of lncRNAs trans-regulatory targeting flowering-associated genes, discerning the potential novel lncRNA- miRNA- mRNA interactions in modulating the lettuce flowering process, and finally via unveiling the relationships between co-expressed lncRNAs and well-studied flowering-related genes via constructing of lncRNA-mRNA co-expression networks. The results of all applied methods together demonstrated that the function of identified lncRNAs in lettuce is mainly promoting/ repressing flowering with or without intermediaries through modulating the master floral regulators including, *FLC*, *CO*, *FT*, and *SOC1*. The findings of this study provided a novel insight into our understanding of lncRNAs’ regulatory functions within the six main flowering-associated pathways in lettuce.

## Methods

### Transcriptomic data

To identify the lncRNAs associated with flowering/bolting in *L. sativa*, two RNA-Seq data sets available in the European Nucleotide Archive (ENA, https://www.ebi.ac.uk/ena) under the BioProject accession numbers PRJNA325073 [9] and PRJNA422994 [3], were downloaded and analyzed. According to the first dataset (PRJNA325073), high throughput RNA-Seq data were used to compare the transcriptomes of seedlings from two near-isogenic lines with different bolting resistance. In the second data set (PRJNA422994), the flowering locus T (*LsFT*) gene was analyzed in 8 developmental stages during the transition from the vegetative to reproductive phase of the same lines. In these studies, a bolting resistant line S24 and a bolting sensitive line S39 were used for RNAs extraction and library construction using NEB Next Ultra Directional RNA Library Prep Kit for Illumina (NEB, Ispawich, MA, United States). Then, RNA-seq libraries were sequenced on an Illumina HiSeq 2000 platform to generate 100 bp pair-ended reads.

### Identification of unannotated transcripts and lncRNAs

For the identification of lncRNAs, the selected data sets were analyzed on the Galaxy website (https://usegalaxy.eu; [124]) using the workflow shown in Fig. S1. In this regard, first, the quality of reads was evaluated by using FastQC (v.0.11.8; https://www.bioinformatics.babraham.ac.uk/projects/fastqc/), and then the low-quality bases and adaptors contamination were eliminated using the Trimmomatic tool (v.0.38; [125]). Pruning the bases with a quality score < Q30 and removing reads with lengths < 50 bp were selected as the most critical Trimmomatic parameters. In the following, high-quality clean reads were mapped to the lettuce reference genome (*L. sativa* cv Salinas V8, https://genomevolution.org/coge/GenomeInfo.pl?gid=28333) by using HISAT2 [24]. The transcripts linked to the reference genome were assembled using StringTie v.2.1.1 [25]. The assembled transcripts were merged with StringTie’s merge tools to make a combined annotation file and re-estimate quantities of transcripts.

To identify unannotated transcripts, transcriptome assemblies (gff files produced by StringTie) were compared with lettuce genome annotation file using the gffcompare program [126]. Then, the unannotated transcripts categorized in the class codes of “u” (intergenic lncRNAs), “x” (antisense lncRNAs), “i” (intronic lncRNAs), “o” (generic exonic overlap lncRNAs with reference transcripts), and “e” (single exon TransFrag overlying a reference exon) were kept as the output results. Since these recognized unannotated transcripts may contain possible coding genes, they were brought under the following multistep filtering scheme:

I. The unannotated transcripts less than 200 bp and greater than 15 Kb CPM (Count per million) < 1 were removed.
II. The result of the former step was entered into tRNAscan-SE 2.0 [127] and later Barrnap 0.9 (https://github.com/tseemann/barrnap) to filter out possible tRNAs and rRNAs.
III. Transcripts with one significant (E-value, 1e-5) hit against the UniProt release 2021–02, Pfam release 34.0, and Rfam 14.5 databases, which encoded a preserved protein/domain were excluded.
IV. The coding potential calculator (CPC2) software [26] was applied to assess the coding potential of foreseen lncRNAs.
V. Finally, CREMA [28] and PLncPRO [27] were employed on the non-coding potential transcripts predicted by CPC2 to increase the accuracy of lncRNA prediction.

### Recognition of differentially expressed mRNAs and lncRNAs

With the help of python script prepDE.py, the differential expression analysis was conducted on the gene read count data matrices. For this analysis, the generated matrices were uploaded onto the IDEAMEX website [128]. After that, DESeq2 [129] software was used to recognize differentially expressed genes (DEGs) and differentially expressed lncRNAs (DE-lncRNA) by screening parameters of FDR ≤ 0.01, log2 fold change (logFC) ≥ 2, and CPM = 1.

### Useful annotation and improvement analysis

For acquiring a functional vision of identifying lncRNAs, a series of approaches were applied which aimed at homology search and characterization of lncRNAs, identification of cis/trans-target genes of lncRNA, and practical enrichment analysis of target genes. Since the distinction between lncRNAs across different classes can provide profound insights into the conservation of lncRNAs, the identified lncRNAs in this study were compared to the lncRNAs reached in Green Non-Coding Database (GREENC; http://greenc.sequentiabiotech.com/wiki/Main_Page; [29]), and Plant Long non-coding RNA Database (PLncDB v.2.0; http://plncdb.tobaccodb.org/; [30]), using the BLASTN tool with the criteria of e-value 1e5, identity more than 70%, and query coverage > 30%.

Afterwards, the role of genes located 100 Kbp upstream and downstream of lncRNAs as cis-regulated potential target genes were investigated through Gene Ontology (GO) and Kyoto Encyclopedia of Genes and Genomes (KEGG) pathway enrichment analysis. Moreover, the untangled connection between lncRNA and DEGs was predicted using LncTar [130] with default parameters.

### Identification of lncRNAs as miRNA endogenous target mimic

LncRNAs can act as miRNA target mimics and negatively adjust and sequester the activity of the miRNAs, owing to the attendance of partial complementarity between lncRNAs and the miRNAs. Hence, using all known lettuce miRNAs, miRNA mimic sites were predicted by psMimic software [31]. Besides, psRobot toolbox [32] was employed to recognize the target genes of the miRNAs that displayed mimicry with lncRNAs. For this analysis, the following parameters were set: penalty score threshold = 2.5, five prime (5’) boundary of essential sequence = 2, three prime (3’) boundary of essential sequence = 17, the maximal number of permitted gaps = 1, and position after which with gap/bulge permit = 17.

### Plant materials and validation by real-time PCR

To confirm the expression levels of predicted lncRNAs, the leafy lettuce genotypes Qomi (early bolting) and Sefid-e-Neishabour (late bolting) were selected according to the results of previous research [33]. To obtain these samples, permission was not necessary. Seeds of genotypes were sown in plastic pots (15 cm diameter, 25 cm high) with sterilized soil and grown in a growth chamber under spring growth conditions for 3 months at Isfahan University of Technology, Isfahan, Iran, in March 2020. Water management was done according to standard practices. The formal identification of the plant material was undertaken by the herbarium of Agricultural and Natural Resources College, University of Tehran, and no voucher specimens were collected and deposited in the collection (it is not necessary as we don’t describe a novel species). We also stated that the field studies were in compliance with local legislation of Iran in the experimental greenhouse and growth chamber of Isfahan University of Technology, Isfahan, and no specific licenses were required.

For this experiment, plants were examined for bolting at 10, 20, 30, and 40 days after planting (Fig. S4) using shoot apical meristem tissues (QDS and SNDS). Sampling from two genotypes was also conducted when the lettuce plants developed into the flowering stage (QFS and SNFS). Samples from 5 different seedlings were pooled together as one biological sample. Three biological replicates from each genotype were used for RNA extraction and the Real-Time PCR (qPCR) analysis according to the previous research [11, 131, 132]. In brief, total RNAs were extracted by the DENAzist column RNA isolation kit. The first-strand cDNA was synthesized using 1 µg of total RNA per sample and RevertAid First Strand cDNA Synthesis Kit (Thermo Fisher, Co., USA), according to the manufacturer’s instructions. Using SYBR Green PCR Master Mix (BioFACT, Korea) and specific primers (Table 2), the Real-Time PCR (qPCR) reaction was performed in a final volume of 15 µl on the ABI system with three technical replicates per each biological replicate (ABI ViiA 7 Real-time PCR). The qPCR program included a single step of initial denaturation at 95 °C for 10 s, followed by 40 cycles of 95 °C for 5 s and 60 °C for 20 s. PP2A1 and TIP41 [133] housekeeping genes were used as internal reference genes to normalize the expression data. Finally, the relative expression levels of lncRNAs were calculated using the 2-ΔΔCt method [134].

**Table 2:** Primers used in quantitative real-time PCR to verify transcriptome sequencing data

Primer	Sequence	Length	Tm	Product Size
MSTRG.45132.1_F	TGACACGAGGATCATACTTTCG	22	60	228
MSTRG.45132.1_R	TACACATGAAGCCTTGTGATCC	22		
MSTRG.78269.1_F	TCAACATACCTCAGAAGGCTCA	22	60	210
MSTRG.78269.1_R	TTGGGTCACAATACTTTCAACG	22		
XR_002846902.1_F	TGACAATCGTTTTCTCACCAAC	22	60	204
XR_002846902.1_R	ATCCATCGTTCGACATTTTACC	22		
XR_002851856.1_F	CTAACTTAATGCTGCCCAGTTG	22	60	141
XR_002851856.1_R	AGCAACAGCAGAAAAATGAGAG	22		
XR_002852627.1_F	TGCTACCGATGTCGAGTGATAC	22	60	205
XR_002852627.1_R	CTGCGTTCATCAGAAGTACCTG	22		
XR_002854255.1_F	GAGGTTAAAGCACCAACGAATC	22	60	171
XR_002854255.1_R	ACGGTCCAAAATGTTTAAGCTC	22		
XR_002852870.1_F	GACTCCATCCATCTCTCGATTC	22	60	155
XR_002852870.1_R	CAACAGGAGTGCCGATATGTAA	22		
XR_002860308.1_F	CGCTGATTATTACGCTGTCATT	22	59.5	193
XR_002860308.1_R	AGTGTGTACGGATCATGCTGAC	22		
XR_002850851.1_F	TCGTTGTTGGCTTCATTATCAC	22	60	225
XR_002850851.1_R	CGTGGTTATTGGTTTGATGTTG	22		
LsPP2A1_F	ATTCATGGTCAATTCTACGATCTGGT	26	60	107
LsPP2A1_R	GAATAATACCCGCGATCAACATAATC	26		
LsTIP41_F	TTTGTATGGAGATGAATTGGCTGATA	26	60	90
LsTIP41_R	CGTAAGAGAAGAAACCAACAGCTAGG	26		

### Co-expression analysis of non-coding/coding genes

For co-expression analysis and identifying modules, methods and parameters were set similar to previous research [132]. In brief, the normalized fragments per kilobase of transcript per million fragments mapped (FPKM) values of DE-lncRNAs and DEGs were entered into WGCNA [135] R package. Pearson’s correlation between the log2 (FPKM + 1) values of all gene pairs was calculated to generate a matrix. Following this, the similarity matrix was transformed into an adjacency matrix. The soft threshold power (β) of 9 was determined based on the scale-free topology standard. Next, the topological overlap measure (TOM) and corresponding dissimilarity (1-TOM) were computed to detect the clusters of densely interconnected genes (modules) and calculate the correlations between modules and the phenotypic trait.

## Supplementary Information

**Additional file 1.**

## Data Availability

All RNA-Seq data were deposited in the NCBI SRA database under the project PRJNA325073 and PRJNA422994 (https://www.ncbi.nlm.nih.gov/bioproject/ PRJNA325073. and https://www.ncbi.nlm.nih.gov/bioproject/ PRJNA422994).
