# Correction: Genome-wide screening and characterization of long noncoding RNAs involved in fowering/bolting of *Lactuca sativa*

**DOI:** 10.1186/s12870-023-04051-y

**Published:** 2023-01-13

**Authors:** Aboozar Soorni, Marzieh Karimi, Batoul Al Sharif, Khashayar Habibi

**Affiliations:** 1 Department of Biotechnology, College of Agriculture, Isfahan University of Technology, Isfahan, Iran; 2 Zarinshahr, Isfahan Iran

**Correction: BMC Plant Biol 23, 3 (2023)**

**https://doi.org/10.1186/s12870-022-04031-8**

Following the publication of the original article [1], the author identified an error in the affiliations of Batoul Al Sharif and Khashayar Habibi. They should be affiliated to Aff1, Department of Biotechnology, College of Agriculture, Isfahan University of Technology, Isfahan, Iran. The correct authors with their corresponding affiliation numbers are presented below:

- Aboozar Soorni^1*^, Marzieh Karimi^2^ , Batoul Al Sharif^1^ and Khashayar Habibi^1^

The correction does not have any effect on the results or conclusions of the paper. The original article [1] has been corrected.
