# Supplementary material for: Genome-wide screening and characterization of long noncoding RNAs involved in flowering/bolting of Lactuca sativa
Source: BMC Plant Biol. 2023 Jan 2;23:3. doi: 10.1186/s12870-022-04031-8 (PMC9806901; doi:10.1186/s12870-022-04031-8)
Supplement: Supplementary file 1 — Additional file 1. [file 12870_2022_4031_MOESM1_ESM.docx]

**Supplementary Fig.1**


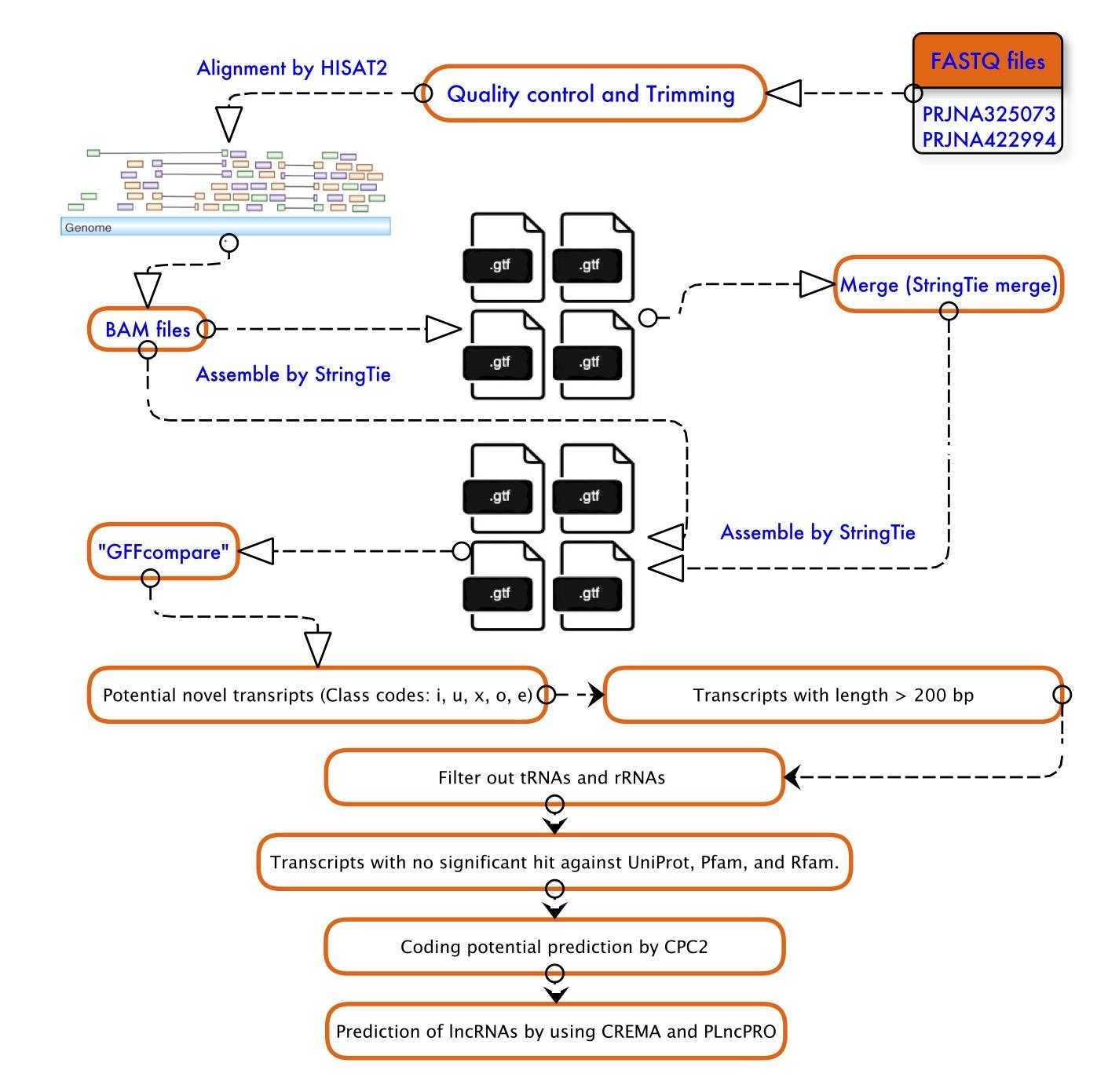


LncRNAs identification pipeline from RNA-Seq data sets.

**Supplementary Fig. 2**


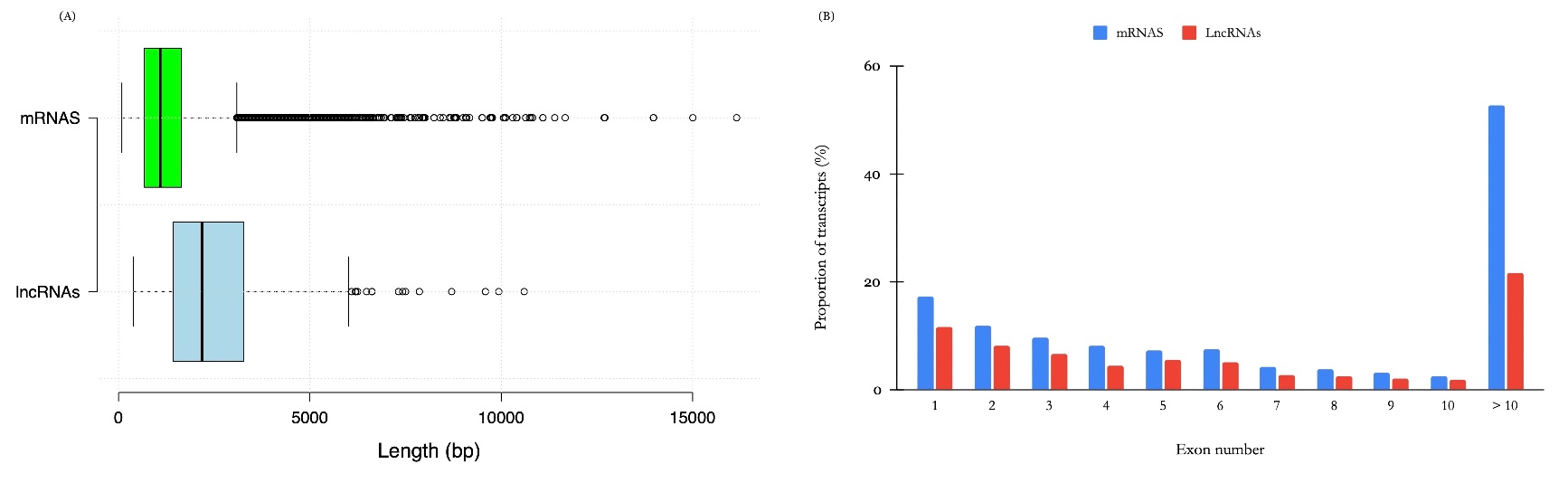


Comparison of lncRNAs and mRNAs by (A) length distribution, and (B) exon number

**Supplementary Table 1 The summarized information related to lncRNAs distribution on the lettuce genome.**

| Scaffold name | Start | Stop | ID |
| --- | --- | --- | --- |
| NW_019672052.1 | 1530576 | 1532207 | MSTRG.259.1 |
| NW_019672057.1 | 294128 | 296522 | MSTRG.456.1 |
| NW_019672057.1 | 306292 | 308613 | MSTRG.459.1 |
| NW_019672058.1 | 837633 | 839053 | MSTRG.554.1 |
| NW_019672058.1 | 967070 | 969425 | MSTRG.571.1 |
| NW_019672062.1 | 575134 | 577478 | MSTRG.676.4 |
| NW_019672063.1 | 695981 | 697103 | MSTRG.751.1 |
| NW_019672065.1 | 78507 | 82074 | MSTRG.900.1 |
| NW_019672072.1 | 53068 | 57085 | MSTRG.1018.2 |
| NW_019672080.1 | 403412 | 405366 | MSTRG.1288.1 |
| NW_019672084.1 | 1044499 | 1045466 | MSTRG.1660.1 |
| NW_019672098.1 | 80848 | 82726 | MSTRG.2401.1 |
| NW_019672099.1 | 63506 | 66470 | MSTRG.2490.1 |
| NW_019672145.1 | 3285121 | 3287780 | MSTRG.3705.1 |
| NW_019672153.1 | 1598804 | 1599942 | MSTRG.3945.2 |
| NW_019672153.1 | 349382 | 352600 | MSTRG.3880.1 |
| NW_019672169.1 | 43443 | 45138 | MSTRG.4044.1 |
| NW_019672186.1 | 497959 | 499652 | MSTRG.4504.1 |
| NW_019672236.1 | 310139 | 314540 | MSTRG.4910.1 |
| NW_019672244.1 | 2556 | 7403 | MSTRG.4939.1 |
| NW_019672251.1 | 1071228 | 1072656 | MSTRG.5020.1 |
| NW_019672257.1 | 81373 | 83892 | MSTRG.5198.1 |
| NW_019672258.1 | 2648737 | 2653305 | MSTRG.5362.1 |
| NW_019672263.1 | 262858 | 265050 | MSTRG.5695.1 |
| NW_019672264.1 | 31496 | 39357 | MSTRG.5724.1 |
| NW_019672358.1 | 832530 | 834808 | MSTRG.6719.1 |
| NW_019672384.1 | 244373 | 245965 | MSTRG.7042.1 |
| NW_019672404.1 | 1225526 | 1227753 | MSTRG.7180.1 |
| NW_019672423.1 | 2143307 | 2144276 | MSTRG.7439.1 |
| NW_019672460.1 | 1135771 | 1136292 | MSTRG.8077.1 |
| NW_019672601.1 | 377620 | 380564 | MSTRG.9400.1 |
| NW_019672626.1 | 1095859 | 1098258 | MSTRG.9853.1 |
| NW_019672689.1 | 542495 | 544956 | MSTRG.10658.1 |
| NW_019672985.1 | 4650195 | 4651788 | MSTRG.14138.1 |
| NW_019672991.1 | 81165 | 84759 | MSTRG.14146.1 |
| NW_019673078.1 | 639481 | 644190 | MSTRG.14955.1 |
| NW_019673148.1 | 1237522 | 1243711 | MSTRG.15422.1 |
| NW_019673281.1 | 2305075 | 2306820 | MSTRG.16505.1 |
| NW_019673296.1 | 1363533 | 1365518 | MSTRG.16744.1 |
| NW_019673358.1 | 146102 | 148089 | MSTRG.17863.1 |
| NW_019673457.1 | 752413 | 753178 | MSTRG.18968.1 |
| NW_019673457.1 | 1417519 | 1418826 | MSTRG.19026.3 |
| NW_019673548.1 | 605442 | 607659 | MSTRG.19699.1 |
| NW_019673724.1 | 1353038 | 1353732 | MSTRG.21854.1 |
| NW_019673724.1 | 4667732 | 4674213 | MSTRG.22037.1 |
| NW_019673733.1 | 2202199 | 2206087 | MSTRG.22394.1 |
| NW_019673747.1 | 208187 | 213517 | MSTRG.22775.1 |
| NW_019673800.1 | 325987 | 327822 | MSTRG.23331.1 |
| NW_019673886.1 | 599804 | 604121 | MSTRG.24072.1 |
| NW_019673944.1 | 2160238 | 2161506 | MSTRG.24590.1 |
| NW_019673970.1 | 341873 | 345814 | MSTRG.25160.1 |
| NW_019673973.1 | 3857 | 5514 | MSTRG.25345.1 |
| NW_019674009.1 | 242585 | 243839 | MSTRG.26018.1 |
| NW_019674009.1 | 876113 | 877681 | MSTRG.26065.1 |
| NW_019674017.1 | 608371 | 611712 | MSTRG.26172.1 |
| NW_019674067.1 | 815056 | 816858 | MSTRG.26389.1 |
| NW_019674087.1 | 31773 | 33992 | MSTRG.26755.1 |
| NW_019674116.1 | 1305710 | 1310805 | MSTRG.27346.1 |
| NW_019674170.1 | 402056 | 403721 | MSTRG.28038.1 |
| NW_019674170.1 | 1446087 | 1446679 | MSTRG.28130.1 |
| NW_019674178.1 | 348315 | 349927 | MSTRG.28376.1 |
| NW_019674178.1 | 350365 | 353963 | MSTRG.28377.1 |
| NW_019674200.1 | 612249 | 615477 | MSTRG.28548.1 |
| NW_019674203.1 | 1074963 | 1077620 | MSTRG.28642.1 |
| NW_019674203.1 | 398634 | 401909 | MSTRG.28595.1 |
| NW_019674205.1 | 182877 | 184870 | MSTRG.28848.1 |
| NW_019674219.1 | 1009940 | 1010333 | MSTRG.29010.1 |
| NW_019674223.1 | 3502850 | 3505060 | MSTRG.29259.1 |
| NW_019674226.1 | 26798 | 28387 | MSTRG.29316.1 |
| NW_019674358.1 | 1418209 | 1422939 | MSTRG.30586.1 |
| NW_019674368.1 | 120423 | 123569 | MSTRG.30852.1 |
| NW_019674382.1 | 159109 | 164507 | MSTRG.31258.1 |
| NW_019674383.1 | 696467 | 703967 | MSTRG.31431.1 |
| NW_019674402.1 | 1540183 | 1546806 | MSTRG.31850.1 |
| NW_019674448.1 | 2124711 | 2126271 | MSTRG.32601.1 |
| NW_019674459.1 | 13606 | 14228 | MSTRG.32649.1 |
| NW_019674470.1 | 138857 | 140956 | MSTRG.32690.1 |
| NW_019674508.1 | 960102 | 962352 | MSTRG.33200.1 |
| NW_019674589.1 | 99213 | 100531 | MSTRG.33872.1 |
| NW_019674705.1 | 152246 | 154751 | MSTRG.34876.1 |
| NW_019674788.1 | 146018 | 152109 | MSTRG.35607.1 |
| NW_019674827.1 | 1832896 | 1833913 | MSTRG.35773.1 |
| NW_019674873.1 | 1231871 | 1234047 | MSTRG.36265.1 |
| NW_019674879.1 | 309211 | 311490 | MSTRG.36477.1 |
| NW_019674883.1 | 1248651 | 1249424 | MSTRG.36596.1 |
| NW_019674927.1 | 188112 | 189513 | MSTRG.36875.1 |
| NW_019674948.1 | 101454 | 105601 | MSTRG.37150.1 |
| NW_019674954.1 | 264419 | 266703 | MSTRG.37181.1 |
| NW_019675027.1 | 595668 | 598947 | MSTRG.37976.1 |
| NW_019675059.1 | 588571 | 589659 | MSTRG.38181.1 |
| NW_019675078.1 | 962724 | 964586 | MSTRG.38466.1 |
| NW_019675078.1 | 1447425 | 1449906 | MSTRG.38506.1 |
| NW_019675088.1 | 295913 | 297148 | MSTRG.38639.1 |
| NW_019675109.1 | 157224 | 158351 | MSTRG.38816.1 |
| NW_019675109.1 | 182045 | 184363 | MSTRG.38820.1 |
| NW_019675109.1 | 219848 | 221368 | MSTRG.38825.3 |
| NW_019675142.1 | 177877 | 181030 | MSTRG.39158.1 |
| NW_019675302.1 | 2907485 | 2908756 | MSTRG.40201.1 |
| NW_019675342.1 | 553100 | 554182 | MSTRG.40600.1 |
| NW_019675592.1 | 983477 | 985282 | MSTRG.42302.1 |
| NW_019675604.1 | 676934 | 677460 | MSTRG.42367.1 |
| NW_019675719.1 | 642043 | 643006 | MSTRG.43145.1 |
| NW_019675719.1 | 649190 | 651033 | MSTRG.43146.1 |
| NW_019675719.1 | 2520849 | 2522826 | MSTRG.43261.1 |
| NW_019675719.1 | 7337661 | 7342404 | MSTRG.43562.1 |
| NW_019675719.1 | 8433170 | 8436000 | MSTRG.43622.1 |
| NW_019675719.1 | 8857028 | 8860430 | MSTRG.43645.1 |
| NW_019675719.1 | 11725032 | 11729967 | MSTRG.43868.1 |
| NW_019675719.1 | 11841514 | 11843015 | MSTRG.43885.1 |
| NW_019675847.1 | 401352 | 402212 | MSTRG.45089.1 |
| NW_019675877.1 | 223779 | 225360 | MSTRG.45132.1 |
| NW_019675877.1 | 852335 | 854138 | MSTRG.45192.1 |
| NW_019675989.1 | 2736114 | 2736742 | MSTRG.46018.1 |
| NW_019676083.1 | 64160 | 65837 | MSTRG.46820.1 |
| NW_019676310.1 | 1793782 | 1803717 | MSTRG.48553.1 |
| NW_019676311.1 | 1860531 | 1863771 | MSTRG.48742.1 |
| NW_019676409.1 | 2723797 | 2725255 | MSTRG.49634.1 |
| NW_019676425.1 | 93123 | 94505 | MSTRG.49751.1 |
| NW_019676438.1 | 1499780 | 1501540 | MSTRG.49911.1 |
| NW_019676537.1 | 1848446 | 1851011 | MSTRG.50915.1 |
| NW_019676572.1 | 3050252 | 3051663 | MSTRG.51545.1 |
| NW_019676667.1 | 46298 | 48499 | MSTRG.52423.1 |
| NW_019676738.1 | 4705782 | 4707245 | MSTRG.53093.1 |
| NW_019676738.1 | 6245395 | 6246908 | MSTRG.53182.1 |
| NW_019676740.1 | 2159979 | 2162914 | MSTRG.53376.1 |
| NW_019676761.1 | 3814106 | 3816226 | MSTRG.53979.1 |
| NW_019676788.1 | 279028 | 285649 | MSTRG.54643.2 |
| NW_019676795.1 | 42603 | 46230 | MSTRG.54732.1 |
| NW_019676795.1 | 67593 | 69304 | MSTRG.54738.1 |
| NW_019676816.1 | 338967 | 340044 | MSTRG.55058.1 |
| NW_019676862.1 | 370809 | 374946 | MSTRG.55550.1 |
| NW_019676919.1 | 223887 | 226067 | MSTRG.56060.1 |
| NW_019676962.1 | 429371 | 430921 | MSTRG.56402.1 |
| NW_019677008.1 | 809996 | 811498 | MSTRG.56741.1 |
| NW_019677035.1 | 2122948 | 2124027 | MSTRG.57390.1 |
| NW_019677106.1 | 2350180 | 2351381 | MSTRG.58354.1 |
| NW_019677106.1 | 4368157 | 4369629 | MSTRG.58497.1 |
| NW_019677106.1 | 1882883 | 1885677 | MSTRG.58303.1 |
| NW_019677158.1 | 3468450 | 3470923 | MSTRG.59177.1 |
| NW_019677158.1 | 96811 | 98006 | MSTRG.58928.1 |
| NW_019677158.1 | 2578990 | 2582207 | MSTRG.59114.5 |
| NW_019677158.1 | 2842033 | 2843623 | MSTRG.59136.2 |
| NW_019677212.1 | 1460183 | 1464472 | MSTRG.60145.1 |
| NW_019677213.1 | 735587 | 738693 | MSTRG.60298.1 |
| NW_019677217.1 | 1641736 | 1642966 | MSTRG.60486.1 |
| NW_019677217.1 | 3435809 | 3443233 | MSTRG.60602.1 |
| NW_019677217.1 | 4765914 | 4768042 | MSTRG.60714.1 |
| NW_019677229.1 | 2188599 | 2190601 | MSTRG.60874.1 |
| NW_019677460.1 | 3000352 | 3002166 | MSTRG.62698.1 |
| NW_019677525.1 | 218352 | 219334 | MSTRG.63517.1 |
| NW_019677629.1 | 1023575 | 1024376 | MSTRG.64254.1 |
| NW_019677658.1 | 1505529 | 1507351 | MSTRG.64822.1 |
| NW_019677673.1 | 233507 | 235384 | MSTRG.64947.1 |
| NW_019677698.1 | 52994 | 58351 | MSTRG.65083.1 |
| NW_019677708.1 | 2588713 | 2589895 | MSTRG.65319.1 |
| NW_019677887.1 | 2948903 | 2951479 | MSTRG.67738.1 |
| NW_019677896.1 | 519459 | 523500 | MSTRG.67790.1 |
| NW_019677904.1 | 744238 | 745666 | MSTRG.68000.1 |
| NW_019677932.1 | 45524 | 48580 | MSTRG.68189.1 |
| NW_019677967.1 | 614871 | 616724 | MSTRG.68580.1 |
| NW_019677967.1 | 646401 | 651119 | MSTRG.68581.1 |
| NW_019677991.1 | 2006 | 5530 | MSTRG.68867.1 |
| NW_019678082.1 | 4401082 | 4405042 | MSTRG.70204.1 |
| NW_019678118.1 | 3005105 | 3008847 | MSTRG.70797.1 |
| NW_019678120.1 | 1907152 | 1908980 | MSTRG.71061.1 |
| NW_019678123.1 | 2692943 | 2695953 | MSTRG.71202.1 |
| NW_019678134.1 | 774044 | 778537 | MSTRG.71492.1 |
| NW_019678143.1 | 295142 | 297663 | MSTRG.71628.1 |
| NW_019678202.1 | 357301 | 358805 | MSTRG.72407.1 |
| NW_019678354.1 | 2068845 | 2071386 | MSTRG.73430.1 |
| NW_019678463.1 | 77862 | 79754 | MSTRG.75255.1 |
| NW_019678489.1 | 2017177 | 2017677 | MSTRG.75449.1 |
| NW_019678502.1 | 335731 | 337665 | MSTRG.75488.1 |
| NW_019678520.1 | 921214 | 931814 | MSTRG.75698.4 |
| NW_019678534.1 | 1109723 | 1112724 | MSTRG.75866.1 |
| NW_019678557.1 | 422873 | 426212 | MSTRG.75944.1 |
| NW_019678681.1 | 880481 | 887798 | MSTRG.76994.1 |
| NW_019678756.1 | 2541827 | 2542461 | MSTRG.77959.1 |
| NW_019678776.1 | 210068 | 212012 | MSTRG.78269.1 |
| NW_019678776.1 | 849673 | 850896 | MSTRG.78306.1 |
| NW_019678836.1 | 9725 | 15346 | MSTRG.78625.1 |
| NW_019678875.1 | 3629925 | 3632316 | MSTRG.79393.1 |
| NW_019678875.1 | 218606 | 221972 | MSTRG.79197.1 |
| NW_019678875.1 | 5179093 | 5182151 | MSTRG.79501.1 |
| NW_019678900.1 | 1333404 | 1339286 | MSTRG.79851.1 |
| NW_019678923.1 | 1050429 | 1051665 | MSTRG.80039.1 |
| NW_019678981.1 | 1475469 | 1476552 | MSTRG.80587.1 |
| NW_019679029.1 | 932510 | 937035 | MSTRG.80963.1 |
| NW_019679077.1 | 1402834 | 1404078 | MSTRG.81920.1 |
| NW_019679123.1 | 830850 | 831981 | MSTRG.82183.1 |
| NW_019679209.1 | 78777 | 80115 | MSTRG.82725.1 |
| NW_019679216.1 | 85879 | 89916 | MSTRG.82811.1 |
| NW_019679269.1 | 78072 | 78851 | MSTRG.83201.1 |
| NW_019679274.1 | 294029 | 296344 | MSTRG.83225.1 |
| NW_019679275.1 | 2764403 | 2764823 | MSTRG.83355.1 |
| NW_019679348.1 | 1150577 | 1151528 | MSTRG.84256.1 |
| NW_019679348.1 | 4601772 | 4603549 | MSTRG.84432.1 |
| NW_019679475.1 | 1154294 | 1155313 | MSTRG.85628.1 |
| NW_019679505.1 | 518672 | 524510 | MSTRG.85709.1 |
| NW_019679602.1 | 2703893 | 2705740 | MSTRG.86863.1 |
| NW_019679602.1 | 2758071 | 2760125 | MSTRG.86867.1 |
| NW_019679650.1 | 2242592 | 2247488 | MSTRG.87252.1 |
| NW_019679688.1 | 136546 | 141212 | MSTRG.87892.1 |
| NW_019679885.1 | 474428 | 479202 | MSTRG.89270.1 |
| NW_019679921.1 | 312333 | 316048 | MSTRG.89670.1 |
| NW_019680002.1 | 1066283 | 1068137 | MSTRG.90586.1 |
| NW_019680019.1 | 2468579 | 2471430 | MSTRG.91158.1 |
| NW_019680027.1 | 2734837 | 2737706 | MSTRG.91364.4 |
| NW_019680027.1 | 1663297 | 1664644 | MSTRG.91294.1 |
| NW_019680029.1 | 425879 | 426415 | MSTRG.91544.1 |
| NW_019680029.1 | 224313 | 230320 | MSTRG.91534.3 |
| NW_019680121.1 | 1670905 | 1673195 | MSTRG.92148.1 |
| NW_019680189.1 | 490678 | 492318 | MSTRG.92877.1 |
| NW_019680189.1 | 1708339 | 1709157 | MSTRG.92965.1 |
| NW_019680212.1 | 1683694 | 1687453 | MSTRG.93452.1 |
| NW_019680318.1 | 780775 | 782822 | MSTRG.94112.1 |
| NW_019680358.1 | 678715 | 680820 | MSTRG.94732.1 |
| NW_019680509.1 | 392139 | 394599 | MSTRG.96156.1 |
| NW_019680555.1 | 64637 | 66573 | MSTRG.96663.1 |
| NW_019680628.1 | 769543 | 772256 | MSTRG.97675.1 |
| NW_019680673.1 | 2015146 | 2019914 | MSTRG.98677.1 |
| NW_019680701.1 | 1734376 | 1738822 | MSTRG.99170.1 |
| NW_019680706.1 | 2244187 | 2247225 | MSTRG.99348.1 |
| NW_019680709.1 | 216192 | 219268 | MSTRG.99452.1 |
| NW_019680780.1 | 1099879 | 1103184 | MSTRG.99920.1 |
| NW_019680820.1 | 986603 | 987170 | MSTRG.100052.1 |
| NW_019680827.1 | 2522314 | 2523093 | MSTRG.100396.4 |
| NW_019680827.1 | 1270306 | 1271833 | MSTRG.100303.1 |
| NW_019680915.1 | 147336 | 149977 | MSTRG.101174.1 |
| NW_019680980.1 | 25288 | 28951 | MSTRG.101983.1 |
| NW_019681034.1 | 1645289 | 1646572 | MSTRG.102479.1 |
| NW_019681142.1 | 365095 | 369439 | MSTRG.104043.1 |
| NW_019681180.1 | 279442 | 281510 | MSTRG.104552.1 |
| NW_019681183.1 | 177318 | 178443 | MSTRG.104567.1 |
| NW_019681216.1 | 497684 | 498670 | MSTRG.105013.1 |
| NW_019681216.1 | 1364745 | 1366246 | MSTRG.105070.1 |
| NW_019681216.1 | 2320038 | 2322117 | MSTRG.105142.1 |
| NW_019681257.1 | 47011 | 49442 | MSTRG.105282.1 |
| NW_019681257.1 | 526775 | 528774 | MSTRG.105322.1 |
| NW_019681371.1 | 306369 | 310319 | MSTRG.106169.1 |
| NW_019681428.1 | 444564 | 449745 | MSTRG.106421.1 |
| NW_019681561.1 | 1881842 | 1887293 | MSTRG.107777.1 |
| NW_019681579.1 | 499532 | 505058 | MSTRG.107930.1 |
| NW_019681595.1 | 158125 | 159020 | MSTRG.107964.1 |
| NW_019681610.1 | 349863 | 351190 | MSTRG.108018.1 |
| NW_019681618.1 | 399878 | 406078 | MSTRG.108105.1 |
| NW_019681700.1 | 277125 | 282109 | MSTRG.108799.1 |
| NW_019681766.1 | 547235 | 548998 | MSTRG.109815.1 |
| NW_019681779.1 | 1196800 | 1199697 | MSTRG.110337.1 |
| NW_019681871.1 | 1621658 | 1622337 | MSTRG.111317.1 |
| NW_019681902.1 | 519568 | 522570 | MSTRG.111752.1 |
| NW_019681902.1 | 524365 | 526683 | MSTRG.111753.1 |
| NW_019681904.1 | 805977 | 815564 | MSTRG.111888.1 |
| NW_019681904.1 | 1921054 | 1923515 | MSTRG.111943.1 |
| NW_019681904.1 | 1921061 | 1923812 | MSTRG.111943.2 |
| NW_019681947.1 | 865015 | 870069 | MSTRG.112529.1 |
| NW_019681958.1 | 99280 | 102199 | MSTRG.112743.1 |
| NW_019682012.1 | 50930 | 51789 | MSTRG.113066.1 |
| NW_019682012.1 | 143163 | 144175 | MSTRG.113070.1 |
| NW_019682175.1 | 66588 | 67716 | MSTRG.114280.1 |
| NW_019682238.1 | 134027 | 136167 | MSTRG.115215.1 |
| NW_019682322.1 | 465261 | 466010 | MSTRG.115633.1 |
| NW_019682406.1 | 190848 | 193622 | MSTRG.116611.1 |
| NW_019682572.1 | 25655 | 27744 | MSTRG.118167.1 |
| NW_019682607.1 | 2223846 | 2226180 | MSTRG.118644.2 |
| NW_019682621.1 | 1100715 | 1106963 | MSTRG.119023.1 |
| NW_019682679.1 | 2744420 | 2745780 | MSTRG.119433.1 |
| NW_019682679.1 | 41915 | 43683 | MSTRG.119241.1 |
| NW_019682834.1 | 51072 | 54322 | MSTRG.120692.1 |
| NW_019682907.1 | 109755 | 112481 | MSTRG.121314.1 |
| NW_019682944.1 | 1889425 | 1892039 | MSTRG.121685.1 |
| NW_019682967.1 | 5131321 | 5132615 | MSTRG.122020.1 |
| NW_019682967.1 | 5838277 | 5839437 | MSTRG.122053.1 |
| NW_019682984.1 | 7116 | 8330 | MSTRG.122159.1 |
| NW_019683062.1 | 790751 | 796489 | MSTRG.122590.1 |
| NW_019683062.1 | 4187344 | 4189594 | MSTRG.122806.1 |
| NW_019683062.1 | 7149606 | 7151405 | MSTRG.122959.1 |
| NW_019683107.1 | 386894 | 389669 | MSTRG.123330.1 |
| NW_019683117.1 | 1821613 | 1830317 | MSTRG.123411.1 |
| NW_019683144.1 | 1852834 | 1854372 | MSTRG.123725.1 |
| NW_019683183.1 | 104949 | 109199 | MSTRG.124227.1 |
| NW_019683183.1 | 136608 | 140454 | MSTRG.124232.1 |
| NW_019683183.1 | 312606 | 313133 | MSTRG.124260.1 |
| NW_019683183.1 | 490063 | 492085 | MSTRG.124276.1 |
| NW_019683231.1 | 188615 | 193621 | MSTRG.125181.1 |
| NW_019683316.1 | 168083 | 170327 | MSTRG.126339.1 |
| NW_019683317.1 | 2298669 | 2300253 | MSTRG.126504.1 |
| NW_019683336.1 | 2660645 | 2661791 | MSTRG.126727.1 |
| NW_019683336.1 | 2660935 | 2661791 | MSTRG.126727.2 |
| NW_019683349.1 | 588777 | 589956 | MSTRG.127048.1 |
| NW_019683394.1 | 2042161 | 2044847 | MSTRG.127404.1 |
| NW_019683401.1 | 699829 | 702127 | MSTRG.127598.1 |
| NW_019683440.1 | 1291593 | 1294864 | MSTRG.127761.1 |

**Supplementary Table 2 The summarized information related to putative flowering-related target genes of miRNAs displayed mimicry with the novel lncRNAs.**

| miRNA | Locus | Name | | Arabidopsis ID | Pathways | Effect on flowering time |
| --- | --- | --- | --- | --- | --- | --- |
| Lsa-miRN1644 | LOC111878276 | PHP, CDC73 | protein CDC73 homolog | AT3G22590.1 | Vernalization | Negative |
| Lsa-miRN1644 | LOC111921608 | CDF2 | cyclic dof factor 1-like | AT5G39660.1 | Photoperiodism | Negative |
| Lsa-miRN1704 | LOC111904861 | LDL2 | lysine-specific histone demethylase 1 | AT3G13682.1 | Autonomous | Positive |
| Lsa-miRN1704 | LOC111911803 | MOS1 | protein MODIFIER OF SNC1 1 | AT4G24680.1 | Autonomous | Positive |
| Lsa-miRN1704 | LOC111909745 | PRP8, SUS2, EMB33 | pre-mRNA-processing-splicing factor 8A | AT1G80070.1 | Autonomous | Positive |
| Lsa-miRN1725 | LOC111880015 | At2-MMP, MMP | metalloendoproteinase 3-MMP-like | AT1G70170.1 | Photoperiodism | Positive |
| Lsa-miRN1725 | LOC111896356 | MRG1 | protein MRG1 | AT4G37280.1 | Autonomous | Positive |

**Supplementary Table 3 The summarized information related to putative flowering-related target genes of miRNAs displayed mimicry with the known lncRNAs.**

| miRNA | Locus | Name | | Arabidopsis ID | Pathways | Effect on flowering time |
| --- | --- | --- | --- | --- | --- | --- |
| Lsa-miRN1644 | LOC111878276 | PHP, CDC73 | protein CDC73 homolog | AT3G22590.1 | Vernalization | Negative |
| Lsa-miRN1644 | LOC111921608 | CDF2 | cyclic dof factor 1-like | AT5G39660.1 | Photoperiodism | Negative |
| Lsa-miRN1704 | LOC111904861 | LDL2 | lysine-specific histone demethylase 1 | AT3G13682.1 | Autonomous | Positive |
| Lsa-miRN1704 | LOC111911803 | MOS1 | protein MODIFIER OF SNC1 1 | AT4G24680.1 | Autonomous | Positive |
| Lsa-miRN1704 | LOC111909745 | PRP8, SUS2, EMB33 | pre-mRNA-processing-splicing factor 8A | AT1G80070.1 | Autonomous | Positive |
| Lsa-miRN1711 | LOC111906141 | DET1, FUS2 | light-mediated development protein DET1 | AT4G10180.1 | Photoperiodism | Negative |
| Lsa-miRN1726 | LOC111896047 | LD | LOW QUALITY PROTEIN: homeobox protein LUMINIDEPENDENS | AT4G02560.1 | Autonomous | Positive |
| Lsa-miRN1728 | LOC111888284 | PRR3 | two-component response regulator-like APRR3 isoform X2 | AT5G60100.1 | Circadian clock | Negative |
| Lsa-miRN4414 | LOC111920961 | ATXR7, SDG25 | histone-lysine N-methyltransferase ATXR7 isoform X1 | AT5G42400.1 | Autonomous | Negative |

**Supplementary Table 4 The number of DE-lncRNAs (novel and known) and flowering related genes in each co-expression module**

| modules | Total | Novel_LncRNAs | Known_LncRNAs | Flowering related genes |
| --- | --- | --- | --- | --- |
| blue | 2224 | 19 | 225 | 26 |
| greenyellow | 117 | 2 | 26 | 0 |
| grey | 956 | 11 | 167 | 11 |
| magenta | 1392 | 5 | 215 | 11 |
| pink | 243 | 8 | 38 | 3 |
| purple | 210 | 4 | 49 | 1 |
| salmon | 101 | 1 | 23 | 2 |
| tan | 115 | 1 | 25 | 1 |

**Supplementary Fig. 3**


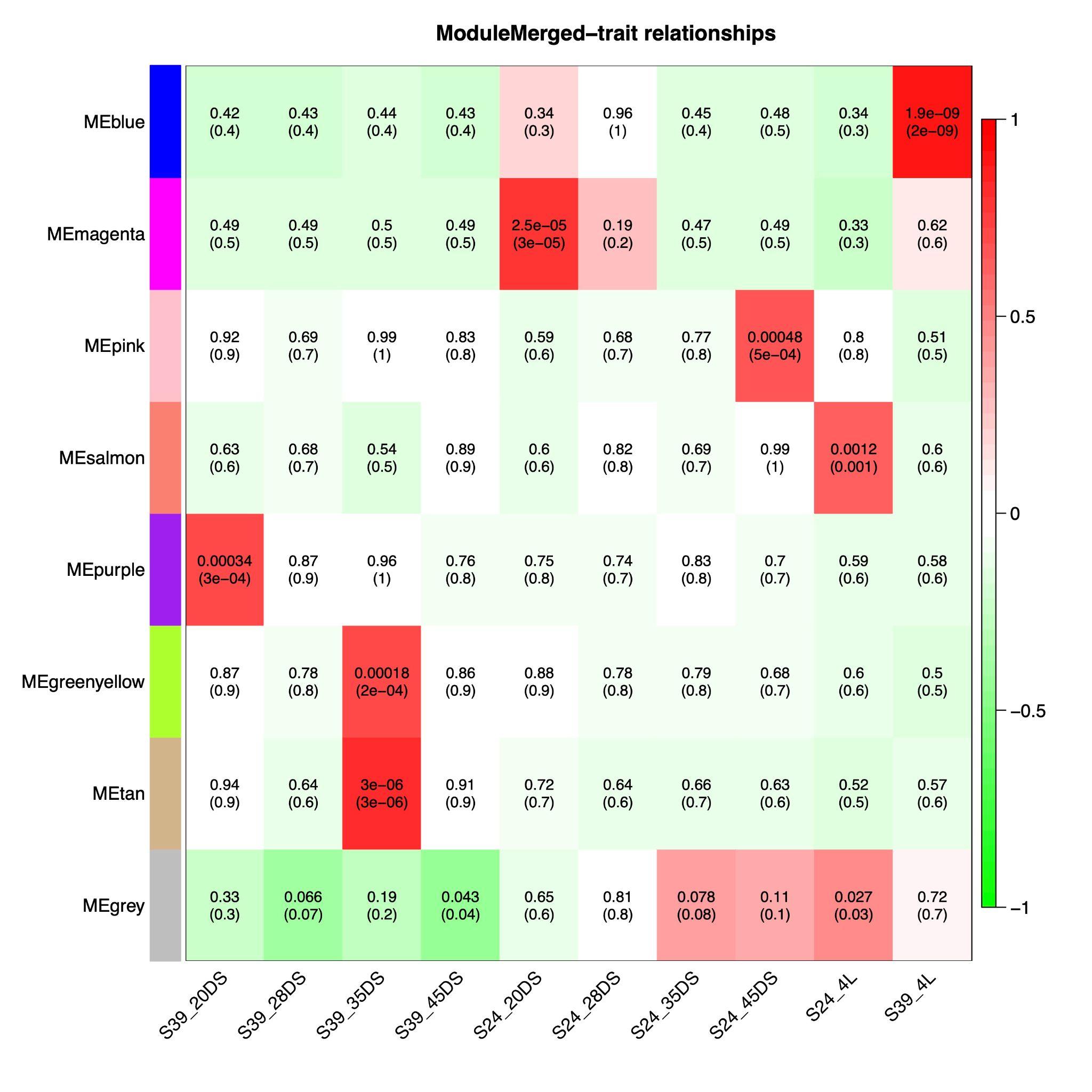


The heatmap of module-traits relationships between different co-expression modules and days after planting traits. Each row and column corresponds to a module eigengene and trait respectively. The numbers represent the Pearson correlation coefficient values and P-values. Green and red colors represent negative and positive correlations, respectively. Color coding of the table was performed by correlation values according to the color legend on the right. 20DS, 28DS, 35DS, and 45 DS represent the days after planting in S24 and S39 lines. 4L represents the fourth-leaf stage.

**Supplementary Fig. 4**


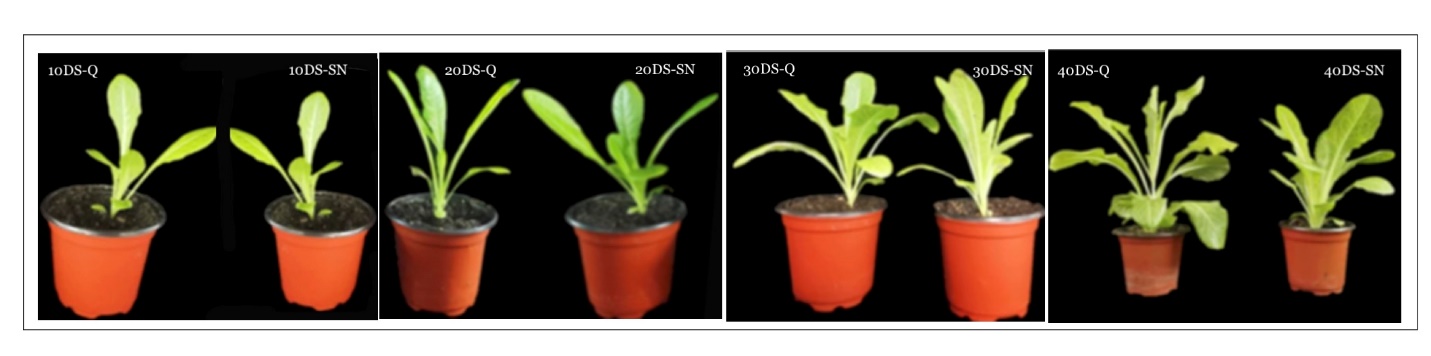


The developmental stages of phenotypes changed on 10, 20, 30 and 40 days after planting.
